# Three Dimensional Structure of the MqsR:MqsA Complex: A Novel TA Pair Comprised of a Toxin Homologous to RelE and an Antitoxin with Unique Properties

**DOI:** 10.1371/journal.ppat.1000706

**Published:** 2009-12-24

**Authors:** Breann L. Brown, Simina Grigoriu, Younghoon Kim, Jennifer M. Arruda, Andrew Davenport, Thomas K. Wood, Wolfgang Peti, Rebecca Page

**Affiliations:** 1 Department of Molecular Pharmacology, Physiology and Biotechnology, Brown University, Providence, Rhode Island, United States of America; 2 Department of Molecular Biology, Cell Biology and Biochemistry, Brown University, Providence, Rhode Island, United States of America; 3 Artie McFerrin Department of Chemical Engineering, Texas A & M University, College Station, Texas, United States of America; 4 Department of Biology, Texas A & M University, College Station, Texas, United States of America; 5 Zachry Department of Civil Engineering, Texas A & M University, College Station, Texas, United States of America; The Rockefeller University, United States of America

## Abstract

One mechanism by which bacteria survive environmental stress is through the formation of bacterial persisters, a sub-population of genetically identical quiescent cells that exhibit multidrug tolerance and are highly enriched in bacterial toxins. Recently, the *Escherichia coli* gene *mqsR* (*b3022*) was identified as the gene most highly upregulated in persisters. Here, we report multiple individual and complex three-dimensional structures of MqsR and its antitoxin MqsA (B3021), which reveal that MqsR:MqsA form a novel toxin:antitoxin (TA) pair. MqsR adopts an α/β fold that is homologous with the RelE/YoeB family of bacterial ribonuclease toxins. MqsA is an elongated dimer that neutralizes MqsR toxicity. As expected for a TA pair, MqsA binds its own promoter. Unexpectedly, it also binds the promoters of genes important for *E. coli* physiology (e.g., *mcbR*, *spy*). Unlike canonical antitoxins, MqsA is also structured throughout its entire sequence, binds zinc and coordinates DNA via its C- and not N-terminal domain. These studies reveal that TA systems, especially the antitoxins, are significantly more diverse than previously recognized and provide new insights into the role of toxins in maintaining the persister state.

## Author Summary

Most bacteria live in biofilms, microbial communities that cause more than 80% of human infections. Biofilms have a genetically identical sub-population of dormant cells, named persister cells, which are the well-recognized source of antibiotic resistance. Recently, it was demonstrated that toxins are highly upregulated in persisters and have therefore been postulated to play a role in the persister state. Using an inter-disciplinary approach, we reveal how *mqsR*, the gene most highly upregulated in persisters, together with *mqsA*, function: they are the founding members of a new family of toxin:antitoxin (TA) systems. Unexpectedly, the structure of MqsR reveals that it is a ribonuclease, a protein that controls the production of other essential proteins. Moreover, we identified multiple features of this TA system that are so unique that each is a starting point for drug development. Unlike other antitoxins, MqsA is structured throughout its entire sequence, its structure is unchanged between the free and toxin-bound states and it binds zinc. It also binds DNA via its C- and not N-terminal domain. Finally, MqsA binds both its own promoter and additional genes important for *E. coli* physiology. Taken together, our data provide fundamental new insights into the role of MqsR and MqsA in bacterial persistence and biofilms.

## Introduction

The emergence of increasing numbers of bacteria that are resistant to antibiotics portends a major public health crisis. One well-recognized but poorly understood mechanism used by bacteria to survive environmental stress is through the formation of persisters, a subpopulation of cells that survive prolonged exposure to antibiotics [1] and exhibit multidrug tolerance [2]. Persisters are not antibiotic-resistant mutants. Instead, they are phenotypic variants that pre-exist in bacterial populations. The dormant, non-dividing persister cells [1]–[3] allow bacteria to survive until the environmental stress is relieved, after which the persisters spontaneously revert to the non-persistent state and repopulate the original culture. Critically, the detailed molecular events that lead to and propagate the persister phenotype are still elusive, as persisters typically represent only a small fraction of the bacterial population. In wild-type *E. coli*, the frequency of persisters in planktonic cultures is only about one in a million [4]. However, in biofilms, complex multicellular bacterial communities that are highly resistant to antibiotics and that are responsible for more than 80% of human infections, this frequency increases substantially, up to one in a hundred [5]. The increased incidence of persister cells in biofilms, and their role in human bacterial infections, has stimulated renewed efforts to understand the molecular mechanism(s) that underlies the persister phenotype.

Recent studies have demonstrated that the persister state is correlated with the increased expression of chromosomal toxins from toxin:antitoxin (TA) genes [2],[6]. TA pairs [7],[8], also known as plasmid addiction systems, are highly abundant on bacterial plasmids [9],[10] and chromosomes [11]–[15]. They are composed of two genes organized in an operon that encode an unstable antitoxin and a stable toxin, respectively. Critical to their function, the protein products of TA pairs have considerable differences in lifetimes [16], with the antitoxin being highly susceptible to degradation by cellular proteases and the toxin comparatively stable. Under normal conditions, the toxin and antitoxin associate to form a tight, non-toxic complex. However, under conditions of stress, the antitoxins are degraded by either the ATP-dependent protease (Lon [16],[17]) or the bacterial protease systems (ClpXP [18]; ClpAP [19]). This leads to a dramatic reduction of both translation and replication rates and, in turn, the cessation of cell growth due to the cellular effects of the toxin. Toxin activities are diverse, and include inhibiting replication by blocking DNA gyrase [20],[21], halting translation via mRNA cleavage [22],[23], or inactivating EF-Tu by phosphorylation [24], among others. TA complexes also typically function as transcriptional repressor:corepressors, where the antitoxin binds to the promoter DNA within the TA operon and the toxin enhances DNA binding [25]–[27].

To date, more than ten TA loci have been identified in *E. coli* [7], including *relBE* [12],[28], *mazEF* [11],[29], *dinJ-yafQ* [30], *hipBA* [24],[31], *hicAB* [32] and *yefM-yoeB* [17],[33]. Gene expression profiling experiments have shown that multiple toxins are highly upregulated in persister cells, especially *relE*, *mazF* and *yoeB* [2],[6]. The activities of these toxins lead to a rapid cessation of cell growth, and have been postulated to play a role in the persistence phenotype [6]. Unexpectedly, the gene most highly upregulated in persisters is *mqsR* (*ygiU*/*b3022*), a gene originally identified as one that encodes a regulator of motility, curli and quorum sensing and that influences biofilm development by mediating the response of the cell to autoinducer-2 [34],[35], but which had not, until recently, been shown to be a bacterial toxin. Because the sequence of MqsR is not similar to that of any other known toxin, its molecular function is unknown.

Deletion of *mqsA* (*ygiT/b3021*), the second gene in the two-gene *mqsRA* operon, is lethal [6],[36]. This led to the postulation that *mqsRA* constitutes a novel TA module [6],[15], with MqsR as the toxin and MqsA as its cognate antitoxin. However, *mqsRA* has many characteristics that differ from canonical TA systems. First, in the *mqsRA* operon, *mqsR* precedes, instead of follows, *mqsA*. This unusual genetic organization has only been observed in two other recently characterized TA systems, that of *higBA* [37] and *hicAB* [32]. Second, their isoelectric points are nearly identical (8.8, MqsR; 9.1, MqsA) rather than being basic and acidic for the toxin and antitoxin, respectively. Third, the MqsA protein is larger, instead of smaller, than MqsR; the only other TA system with an antitoxin larger than its cognate toxin is that of *hicAB* [32]. Finally, their sequences are not homologous to any member of a recognized TA system.

In this paper, we employed a combination of biochemical and structural studies to show that MqsR, along with MqsA, are a *bona fide* TA pair that, because of the unique features of MqsA, define a novel family of TA modules. We show that MqsR is toxic and forms a tight complex with its antitoxin, MqsA, an interaction that mitigates MqsR toxicity. MqsA and the MqsR:MqsA complex also bind the promoters of the *mqsRA* operon and, unexpectedly, genes critical for *E. coli* physiology, including *mcbR* and *spy*. To the best of our knowledge, this is the first time a TA pair has been shown to bind and regulate promoters other than its own. The structure of MqsR reveals that it is a member of the RelE/YoeB family of bacterial RNase toxins. Based on its similarity with RelE, MqsR likely functions as a ribosome-dependent RNase. This suggests that MqsR is important for bacterial persistence via its ability to inhibit translation and, in turn, cell growth. MqsA itself is a two-domain protein with a novel fold that, unlike every other antitoxin, is well-ordered throughout its entire sequence and whose structure does not change upon toxin binding. It is also the first antitoxin known that binds metal, in this case zinc. These studies reveal the molecular mechanisms by which MqsR and MqsA mediate the cessation of cell growth and provide novel targets for the development of a new class of antibiotics that target TA pairs.

## Results

### *mqsRA* is a bona fide TA locus

**Figure 1:**
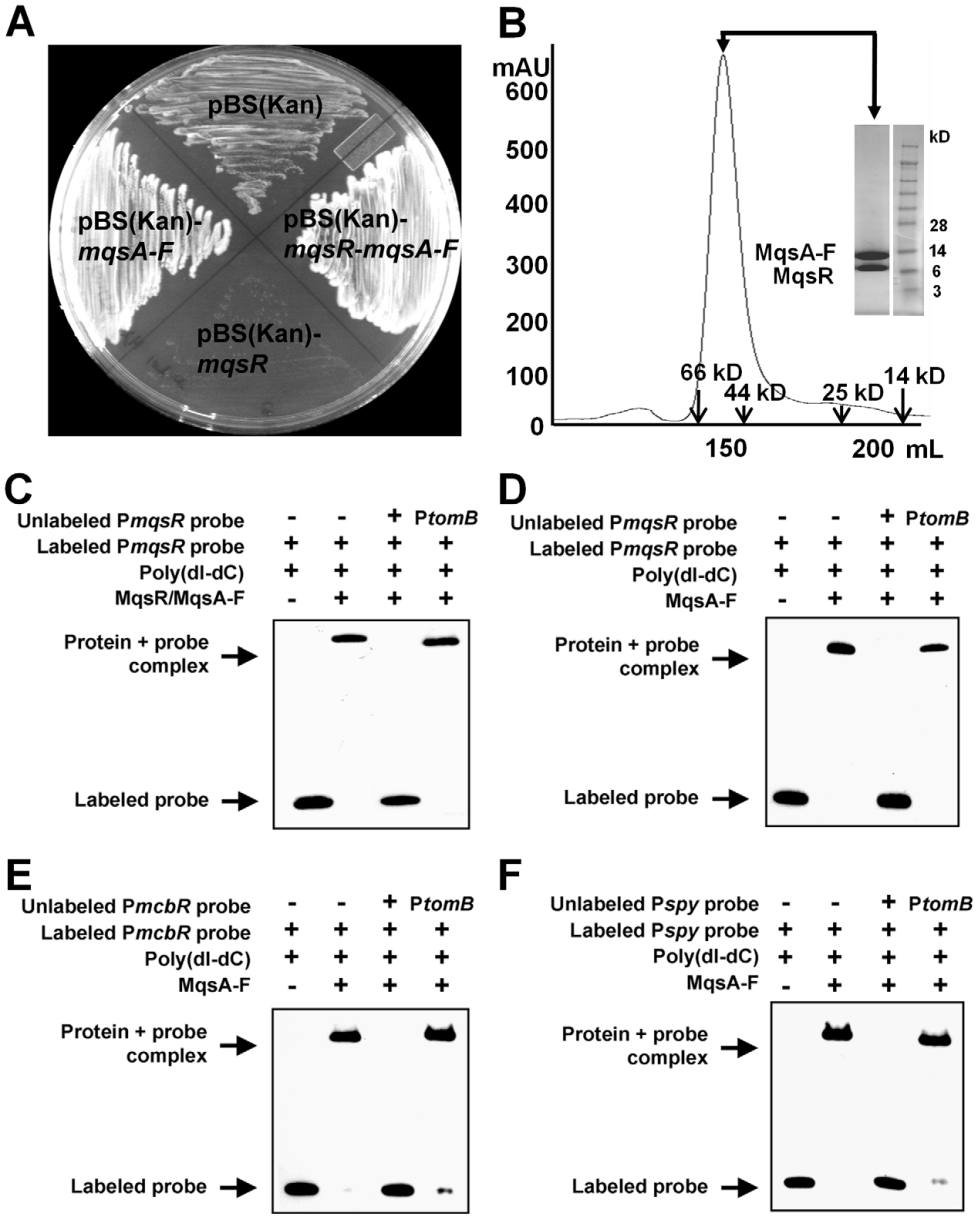
MqsR:MqsA are a *bona fide* toxin:antitoxin pair. (A) The effect of *mqsR*, *mqsA-F* and *mqsR-mqsA-F* on colony formation for *E. coli* strain BW25113 containing pBS(Kan; empty plasmid), pBS(Kan)-*mqsR*, pBS(Kan)-*mqsA-F*, and pBS(Kan)-*mqsR-mqsA-F*. (B) Size exclusion chromatogram and SDS-PAGE gel of the MqsR:MqsA-F complex (Superdex 75 26/60 column; MqsA-F, 14.7 kD; MqsR, 11.5 kD). Elution positions of molecular weight standards are indicated by arrows. The complex elutes as a single peak at a position consistent with the formation of a dimer of dimers (two copies of MqsR and two copies of MqsA-F; expected MW of the complex  = 52.4 kD). Electrophoretic mobility shift assays: both the MqsR:MqsA-F complex (C) and MqsA-F alone (D) bind the *mqsR* promoter; MqsA-F also binds the *mcbR* promoter (E) and the *spy* promoter (F). P*tomB* [61] is a control DNA fragment that we show in Figures S1F, S1G is not bound by either the MqsR:MqsA-F complex or MqsA-F.

The ability of MqsR to arrest cell growth was examined by measuring its effect on colony formation (CFU/ml) and cell viability. Expression of MqsR alone leads to cell growth arrest in multiple bacterial strains (BW25113 and MG1655), while co-expression of MqsR with full-length MqsA (referred to hereafter as MqsA-F) rescues the cell growth arrest phenotype (Figs. 1A, S1A-E and [6],[38]). In addition, MqsR and MqsA-F form a tight oligomeric complex, as MqsA-F (untagged) co-purifies with MqsR (his-tagged) and forms a dimer of dimers, composed of two copies of MqsR and two copies of MqsA-F (hereafter referred to as MqsR:MqsA_2_:MqsR), as determined using size exclusion chromatography (Fig. 1B) and confirmed using dynamic light scattering. Furthermore, deletion of *mqsA* is lethal [6],[36]; similar results have been found with other antitoxins, such as HigA of *Vibrio cholerae* [39]. MqsA-F is also sensitive to proteolysis (Fig. S2). Using electrophoretic mobility shift assays (EMSA), we demonstrate that both the MqsR:MqsA_2_:MqsR complex and MqsA-F bind specifically to the *mqsR* promoter (P*mqsR*; Figs. 1C, 1D). It was also recently shown that MqsA binds two distinct palindromic sequences within P*mqsR* and that MqsA binding is enhanced in the presence of MqsR [38]. Finally, MqsR and MqsA are conserved, both in sequence and in gene structure, throughout the *gamma*- *delta*- and *epsilon* proteobacterial classes (Fig. S3). Taken together, these results show that MqsR is a *bona fide* toxin and MqsA is the proteolytically sensitive antitoxin that blocks MqsR toxicity. Because MqsR:MqsA_2_:MqsR was also recently identified to regulate the expression of a number of *E. coli* genes including one that encodes the colonic acid regulator McbR [34],[40], we reasoned that this regulation may be mediated by the direct binding of MqsA-F and/or the MqsR:MqsA_2_:MqsR complex to the promoters of these genes. EMSA was used to show that MqsA-F binds specifically to the promoters of *mcbR* and *spy* (Figs. 1E, 1F, S1F, S1G).

### Structure determination

**Figure 2:**
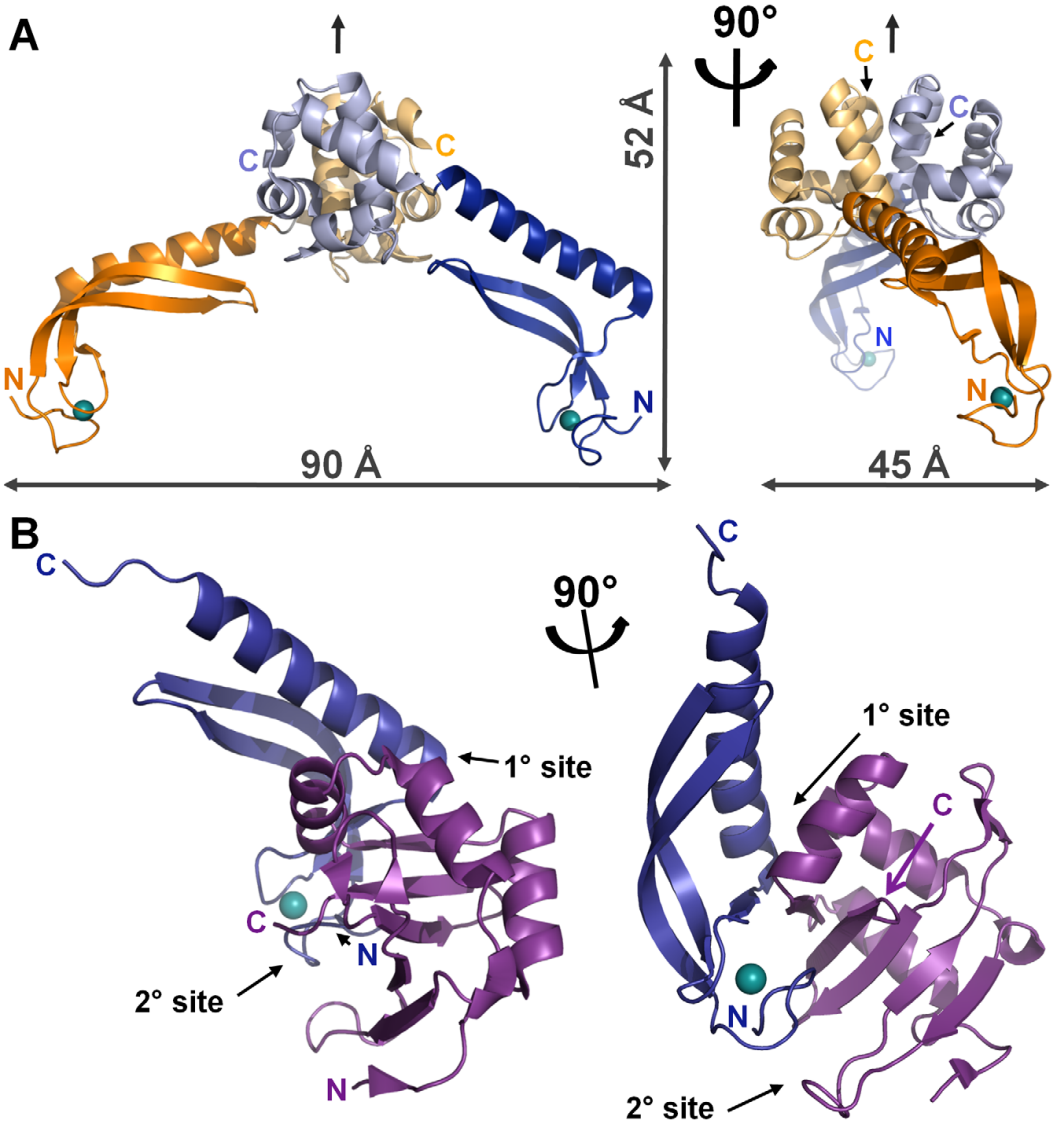
The structures of MqsA and MqsR. (A) Ribbon representation of the MqsA-F dimer. The MqsA-F dimer is composed of two chains (blue and orange), each of which have two distinct N- and C-terminal domains (illustrated in dark and light shades, respectively). MqsA-F dimerization is mediated by the C-terminal HTH-XRE domains (MqsA-C; light blue and light orange), with the N-terminal zinc binding domains (MqsA-N; dark blue and dark orange) projecting away from the dimer interface; the coordinated zinc ion is shown as a teal sphere. The local two-fold axis relating the MqsA-F monomers to one another is indicated by an arrow. The dimensions of the dimer (45×52×90 Å) are shown. (B) Ribbon representation of the complex between MqsR (magenta) and MqsA-N (dark blue). The MqsR:MqsA-N interface is composed of two sites, the primary site (1°) and the secondary site (2°). In (A) and (B), the panel on the right is rotated by 90° with respect to that on the left about the axis shown.

The structure of full-length MqsA (MqsA-F; residues 1–131, the constructs used in this study are shown in Fig. S4) is shown in Figure 2A. MqsA-F was determined to a resolution of 2.15 Å by molecular replacement using the structures of the MqsA N-terminal domain (residues 1–76, referred to hereafter as MqsA-N; Fig. S5A) and the MqsA C-terminal domain (residues 62–131; referred to hereafter as MqsA-C; Fig. S5B), as search models. The space group of the MqsA-F crystal is P2_1_, with two molecules in the asymmetric unit. The two monomers are identical in terms of the overall fold with well-ordered electron density throughout both chains. The final model contains 262 residues, and includes all 131 residues for each MqsA-F molecule.

The structure of the MqsR:MqsA-N complex (Fig. 2B) was solved to a resolution of 2.0 Å using the multiple wavelength anomalous dispersion method. The space group of the crystal is P4_1_2_1_2, with two crystallographically independent complexes in the asymmetric unit. The two complexes are identical in terms of the overall fold of each protein, with well-ordered electron density observed throughout the first complex and throughout the majority of the second complex. The final model contains 70 residues (1–170) of the 76 residues for MqsA-N and 97 residues (1–97) of the 98 residues for MqsR in the first complex and 59 residues (1–20, 27–65) of MqsA-N and 90 residues (2–60, 64–95) for MqsR in the second complex. X-ray diffraction data quality and refinement statistics are reported in Table 1.

**Table 1:** Data collection and refinement statistics for MqsA-F and MqsR:Mqs-N.

	MqsA-F	MqsR:MqsA-N (peak)	MqsR:MqsA-N (inflection)	MqsR:MqsA-N (remote)
**Data Collection** 1				
Space Group	P2_1_		P4_1_ 2_1_ 2	
Unit Cell (Å)	62.1, 31.0, 75.5		63.2, 63.2, 194.9	
Unit Cell (°)	90.0, 106.6, 90.0		90.0, 90.0, 90.0	
Wavelength (Å)	1.0	1.2815	1.283	1.0
Resolution (Å)	50–2.15 (2.19–2.15)	50.0–2.30 (2.38–2.30)	50.0–2.30 (2.38–2.30)	50.0–2.00 (2.03–2.00)
R_sym_ (%)	7.4 (27.1)	4.7 (12.9)	4.8 (13.7)	5.8 (36.5)
<I/σI>	12.9 (4.85)	65.5 (15.1)	66.7 (28.9)	36.0 (8.6)
Completeness (%)	99.4 (99.7)	97.0 (98.1)	96.9 (98.9)	95.8 (98.0)
Redundancy	3.2 (3.1)	6.9 (2.4)	7.6 (7.2)	12.5 (11.6)
**Refinement Statistics**				
Resolution (Å)	50.00–2.15			40.64–2.00
R_cryst_ (%)	18.2			20.5
R_free_ (%)	23.3			24.8
Protein atoms	2052			2495
Waters	159			207
Ions	2			2
r.m.s.d bond length (Å)	0.011			0.013
r.m.s.d bond angle (°)	1.225			1.338
**Average B Factors (Å^2^)**				
Protein	27.31			35.29
Water	29.53			23.10
Ions	23.61			15.79
**Ramachandran Plot**				
Favored (%)	98.8			99.0
Allowed (%)	1.2			1.0
Disallowed (%)	0.0			0.0
**PDB Code**	3GN5			3HI2

1 Highest-resolution shell data are shown in parentheses.

### MqsA is a structured antitoxin

**Figure 3:**
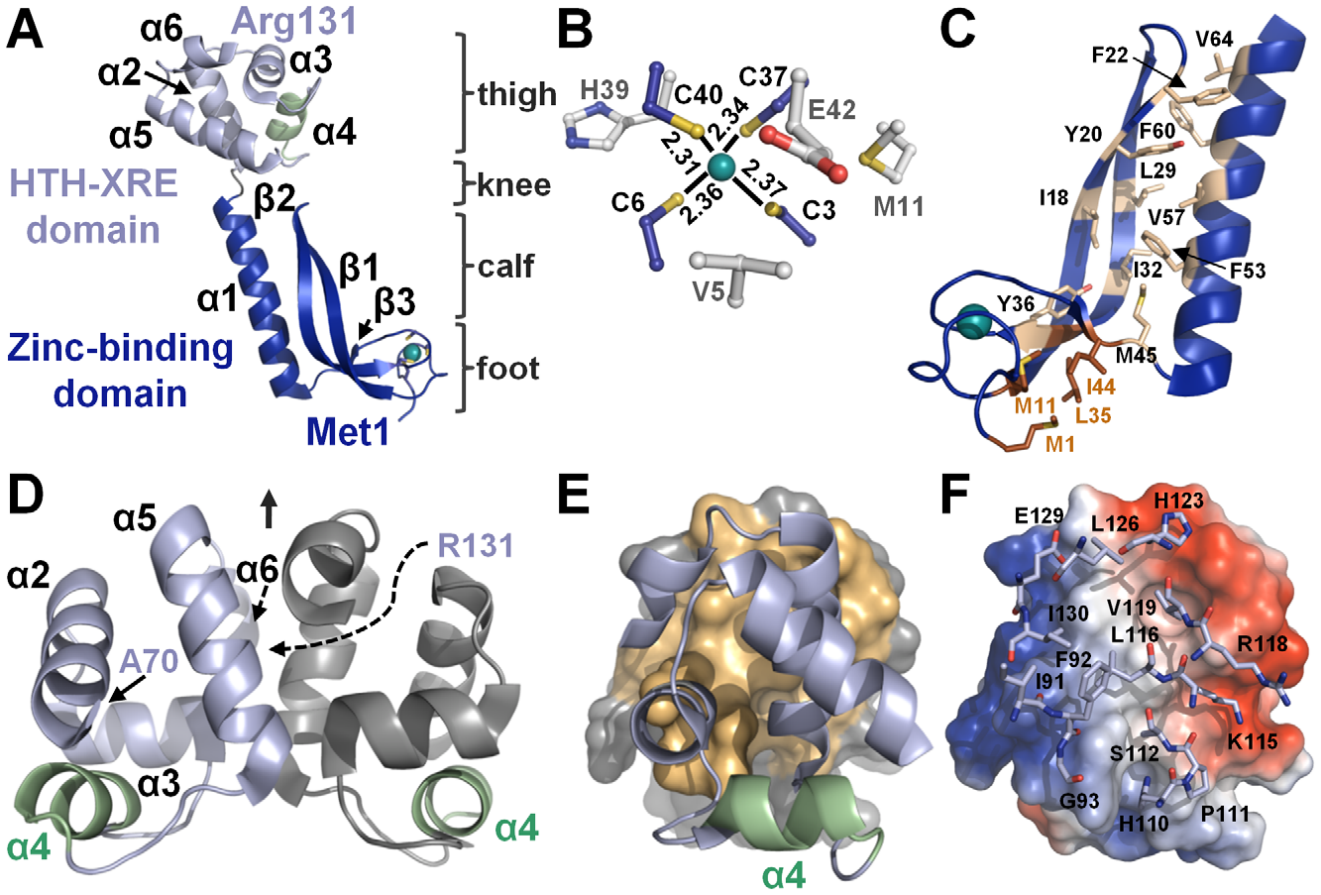
MqsA is a structured antitoxin. (A) The MqsA-F monomer can be visualized as a ‘leg’: MqsA-C (the HTH-XRE domain; light blue) is the thigh, the flexible linker (centered on T68) is the knee and MqsA-N (the zinc binding domain; dark blue) is the ‘calf’ and ‘foot’; the zinc (teal sphere) is bound by the ‘toes’. (B) The zinc is coordinated by Cys3, Cys6, Cys37 and Cys40 (blue/yellow sticks). Sulfur-zinc distances are commensurate with values typical for structural zinc binding sites. Residues near the zinc binding pocket are also shown (grey sticks). (C) The MqsA zinc binding domain adopts a novel fold characterized by a long, twisted β-sheet buttressed by a five-turn α-helix with the loops coordinated by zinc. It is stabilized by two hydrophobic cores (core 1, sidechains shown as sticks in beige; core 2, sidechains shown as sticks in orange). (D) The MqsA-C dimerization interface is composed of the MqsA-C α-helices 3, 5, and 6, with the two monomers (chain A, grey; chain B, light blue) related by local two-fold symmetry. The sequence most highly conserved among MqsA proteins (residues 98–105, green) is predicted to bind DNA (see text). (E) The MqsA-C dimerization interface rotated by 90° with one monomer represented in surface representation (1600 Å^2^ of buried SASA illustrated in beige) and the second in ribbon representation. (F) Residues buried at the MqsA-C dimer interface are shown as sticks (chain B) and the charge distribution is shown in surface representation (chain A; positive charge, blue; negative charge, red).

The structure of the MqsA-F dimer is shown in Figure 2A. The two monomers are related to one another by local two-fold symmetry (Fig. 2A, arrow). The MqsA monomer is composed of two structurally distinct domains connected by a flexible linker and resembles a human leg (Fig. 3A): the MqsA-N, composed of residues 1–67, is the ‘foot’ and ‘calf’ and the MqsA-C, composed of residues 69–131, is the ‘thigh’. The ‘knee’-like linker connecting the domains, centered on residue 68, is flexible, allowing the N- and C-terminal domains to rotate independently of one another as rigid bodies (superposition of the N- and C-terminal domains from chains A and B give RMSD values of 0.35 Å and 0.58 Å, respectively). Superposition of the C-terminal domains from both monomers shows that the corresponding N-terminal domains are rotated by ∼25° with respect to one another (Fig. S5C).

MqsA-N binds zinc and adopts a novel, elongated fold (Figs. 3A–3C). It is composed of one long five-turn α-helix, a twisted β-sheet, loops that connect the secondary structural elements and a coordinated zinc ion. The zinc, which serves a structural and not catalytic role [41],[42], is coordinated by four cysteines (Cys3, Cys6, Cys37, Cys40) with an average sulfur-zinc distance of 2.35 Å (Figs. 3B, S6A). In spite of the low sequence identity (12% identity, 18% similarity) in the MqsA-N domain among different species, these zinc-coordinated cysteines are perfectly conserved (Fig. S3B), suggesting that all bacterial MqsA proteins adopt a similar fold.

The interaction between the two, long twisted β-strands and the five turn α-helix of the MqsA zinc binding domain is stabilized by an extended hydrophobic core composed of 11 residues: Ile18, Tyr20, Phe22, Leu29, Ile32, Tyr36, Met45, Phe53, Val57, Phe60 and Val64 (Fig. 3C; residues in beige). Although none of these residues are identical among the MqsA bacterial homologs (Fig. S3B), they are highly similar. A second cluster of hydrophobic residues is found near the zinc binding pocket and includes Met1, Met11, Leu35 and Ile44 (Fig. 3C; residues in orange). The DALI server [43] was used to identify proteins with similar folds. Although more than 269 hits were obtained (Z-scores from 3.2 to 2.0), they aligned to only the two long β-strands and the five turn α-helix; none had the combination of strands, helix and the zinc binding pocket observed in MqsA. Thus, to the best of our knowledge, MqsA-N is a zinc binding domain with a novel fold, hereafter referred to as the MqsA fold.

MqsA-C, the helix-turn-helix (HTH) domain, is composed of five tightly packed α-helices (Figs. 3A, 3D-3E), which bury a central hydrophobic core. This core is composed of eight residues, including Val69, Val77, Leu83, Phe92, Phe99, Tyr102, Pro109, and Leu117. Of these, all but one (Val69) are perfectly conserved among the MqsA family (Fig. S3B). Structural similarity searches demonstrated that MqsA-C shows significant homology to the bacteriophage 434 Cro repressor, the P22 C2 repressor and HigA antitoxin, placing it in HTH-XRE family of DNA binding proteins [44].

MqsA dimerization is mediated by the MqsA-C HTH-XRE domain. Residues from α-helices 3, 5, and 6 participate in the dimerization interface (Fig. 3D), which buries 1600 Å^2^ of solvent accessible surface area (SASA; this constitutes 19.9% of the total SASA; Fig. 3E) and has a surface complementarity of 0.69, both of which are well within the ranges expected for biologically relevant protein:protein interactions. Six residues are buried upon dimer formation, including Ser112, Lys115, Leu116, Val119, Leu126 and Ile130 (Fig. 3F). Five of these six residues are either identical or highly similar among the MqsA family (Fig. S3B). Eight additional residues (Ile91, Phe92, Gly93, His110, Pro111, Arg118, His123 and Glu129) participate, but are not fully buried, in the dimerization interface. Residues from α-helix 5 (Pro111, Leu116, Val119 and the aliphatic portions of the Lys115 and Arg118 sidechains) form a long hydrophobic pocket down the center of the face of the monomer, which is bordered on one side by negatively charged residues and on the other by positively charged residues (Fig. 3F).

### The MqsA antitoxin binds the MqsR toxin and DNA via distinct domains

**Figure 4:**
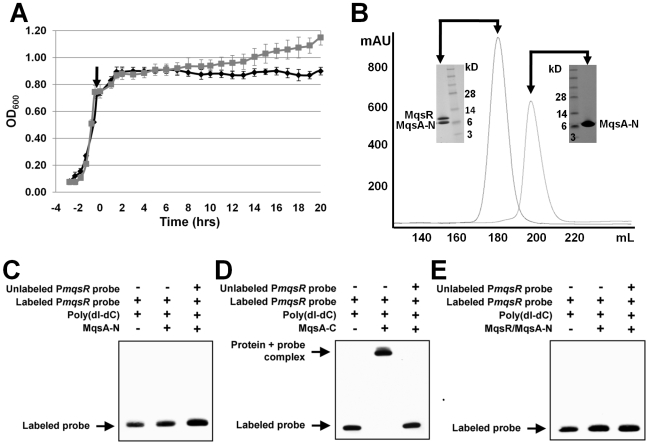
MqsA binds MqsR via its MqsA-N zinc binding domain and DNA via its MqsA-C HTH domain. (A) Growth curves of the co-expression of MqsR with the N-terminal domain of MqsA (MqsR:MqsA-N, grey square) and the C-terminal domain of MqsA (MqsR:MqsA-C, black diamond) in BL21 (DE3) cells. Induction of protein expression using 0.5 mM IPTG corresponds to t = 0 (arrow; expression carried out at 18°C, following the same protocol used to produce the proteins for structural studies). Co-expression of MqsR with MqsA-C leads to growth arrest while co-expression with MqsA-N results in robust growth. Data shown represents the average of the two measurements with the standard deviation shown as error bars. (B) Size exclusion chromatogram (SEC) of the co-expressed MqsR:MqsA-N complex (dark grey, left) and the SDS-PAGE gel of the pooled peak. As can be seen, both MqsR (11.5 kD) and MqsA-N (8.5 kD) are present. The MqsR:MqsA-N SEC, with corresponding SDS-PAGE gel, is overlaid with that of MqsA-N alone (light grey, right), illustrating the shift in the elution position of MqsA-N upon complex formation. (C–E) EMSAs using only MqsA-N (C), only MqsA-C (D) or the MqsR:MqsA-N complex (E) with the *mqsR* promoter.

In order to determine which domain of MqsA (MqsA-N, MqsA-C or both) binds MqsR, we carried out two co-expression toxicity experiments using the same protocol as that used to produce the proteins for structural studies: 1) MqsR co-expressed with MqsA-N and 2) MqsR co-expressed with MqsA-C. When MqsR and MqsA-C are co-expressed, bacterial cell growth is arrested (Figs. 4A, S7A, black diamond). In contrast, when MqsR and MqsA-N are co-expressed, cell growth is robust and both MqsR and MqsA-N express to high levels (Figs. 4A, S7A, grey square). Moreover, following co-expression, the MqsR:MqsA-N complex is readily purified and forms a heterodimer (one copy of MqsR and one copy of MqsA-N), as determined using size exclusion chromatography (Fig. 4B), and confirmed using dynamic light scattering.

EMSA was used to determine which domain of MqsA binds DNA. As shown in Figures 4C–E, the MqsA C-terminal domain is necessary and sufficient for binding the *mqsR* promoter (P*mqsR*), as incubation of P*mqsR* with MqsA-C results in a shift in the electrophoretic mobility of the DNA (Fig. 4D). In contrast, no shift is observed when the DNA is incubated with either MqsA-N alone or the MqsR:MqsA-N complex (Figs. 4C, 4E). These results show that DNA binding is mediated exclusively by MqsA-C.

### MqsR adopts an RNase fold characteristic of the YoeB, RelE toxin family

**Figure 5:**
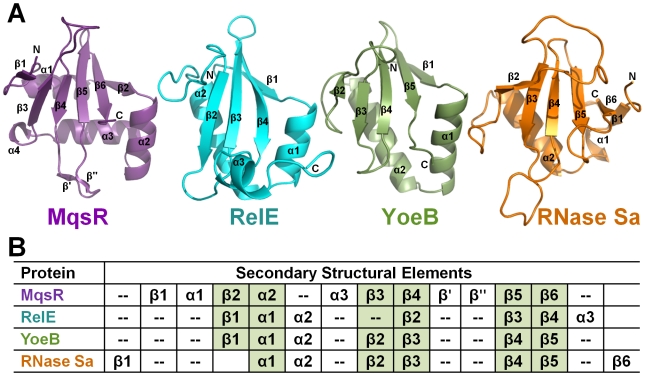
MqsR shares structural homology with the RelE and YoeB family of *E. coli* RNase toxins. (A) MqsR (magenta) with labeled secondary structural elements, and those of its closest structural homologs YoeB (green; DALI Z-score = 5.1; PDBID 2A6Q [33]) and RelE (cyan, DALI Z-score = 4.0; PDBID 2KC8 [45]). RNase Sa (orange; PDBID 1RSN [48]), a canonical bacterial RNase, is also shown. The bacterial RNase fold is characterized by a central, twisted β-sheet and adjacent α–helix and is conserved among all four proteins. (B) Corresponding secondary structural elements of each protein; elements highlighted in green are structurally conserved between the proteins.

MqsR is a small, globular protein, consisting of a central six-stranded β-sheet (β1-β3-β4-β5-β6-β2) and three α-helices, with α-helix 2 adjacent to and α-helices 1 and 3 abutting the backside of the β-sheet (Fig. 5A, left; magenta). A three-dimensional structure alignment revealed that MqsR is most similar to the bacterial toxins YoeB [33] and RelE/aRelE [28],[45] (DALI Z-scores  = 5.1 and 4.0/5.3 respectively [43]), both of which are ribonucleases (RNases) and adopt a microbial RNase fold, the RelE-like fold [46] (Fig. 5A; PDBIDs: RelE, 2KC8; YoeB, 2A6Q; RNase Sa, 1RSN). The sequence identity of MqsR with YoeB and RelE/aRelE is extremely low, only 11% and 13%, respectively, and demonstrates why structure determination was essential to identify MqsR as a member of this family. Critically, our finding that MqsR functions as a bacterial ribonuclease toxin was recently confirmed [38].

The RelE-like fold is characterized by a central, antiparallel β-sheet and adjacent α-helix, which is conserved among the MqsR, YoeB and RelE bacterial toxins (Fig. 5B; conserved secondary structural elements shaded in green). However, there are a few key differences. First, the β-sheet in MqsR is extended by one β-strand (β1) because MqsR has a longer N-terminus. Second, in contrast to YoeB, RelE and RNase Sa, which each have one α-helix that folds across the back of the central β-sheet at a 45° angle, MqsR has two α-helices, both of which are perpendicular to the β-strands. This allows the long loop in MqsR that connects β-strands 4 and 5 to extend towards and interact with α-helix 2, an interaction that is sterically prohibited in YoeB and RelE.

### MqsR potential functional site

**Figure 6:**
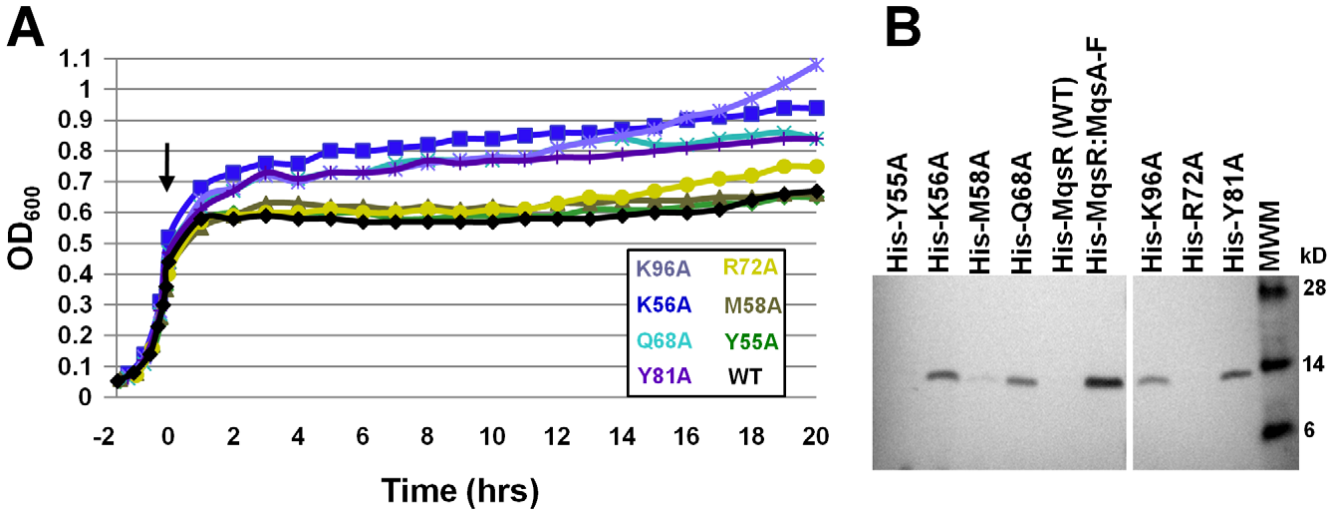
Identification of the MqsR functional site. (A) Growth curves of cells over-expressing WT MqsR and seven MqsR mutants. MqsR mutants K56A, Q68A, Y81A and K96A show decreased toxicity compared to WT MqsR, as evidenced by their ability to grow following induction with 0.5 mM IPTG at t = 0 (arrow; expression carried out at 18°C, following the same protocol used to produce the proteins for structural studies). In contrast, induction of MqsR mutants Y55A, M58A and R72A, like WT, lead to growth arrest. (B) Western blots showing soluble expression of MqsR mutants K56A, Q68A, Y81A and K96A from the cultures used to generate the growth curves in (A) (WT MqsR arrests cell growth so rapidly that free MqsR is not detectable by Western Blot). All MqsR constructs include an N-terminal his_6_-tag and protein expression was detected using a polyhistidine polyclonal antibody. Like WT, expressed protein for MqsR mutants Y55A, M58A and R72A was not detected.

In order to test which residues play a role in MqsR-mediated toxicity, we used alanine-scanning mutagenesis of evolutionarily and structurally conserved residues. Toxicity was measured by monitoring MqsR-mediated growth arrest and protein expression levels. The MqsR mutants that exhibited the most robust growth (i.e., those with the least toxicity) were K56A, Q68A, Y81A and K96A (Figs. 6A, S7B). The mutant proteins also expressed and could be detected by Western Blot using an antibody directed to the MqsR his_6_-tag (expression of wildtype, WT, MqsR arrests cell growth so rapidly that free MqsR is not detectable, even by Western Blot; Fig. 6B). In contrast, growth curves for MqsR mutants Y55A, M58A and R72A were similar to WT and, like WT, did not express to detectable levels. This was also observed for MqsR mutants H7A, H64A and H88A (not shown). Thus, these results show that MqsR residues K56, Q68, Y81 and K96 play key roles in MqsR-mediated toxicity while residues H7, Y55, M58, H64, R72 and H88 do not.

### MqsA recognition and neutralization of the MqsR toxin

**Figure 7:**
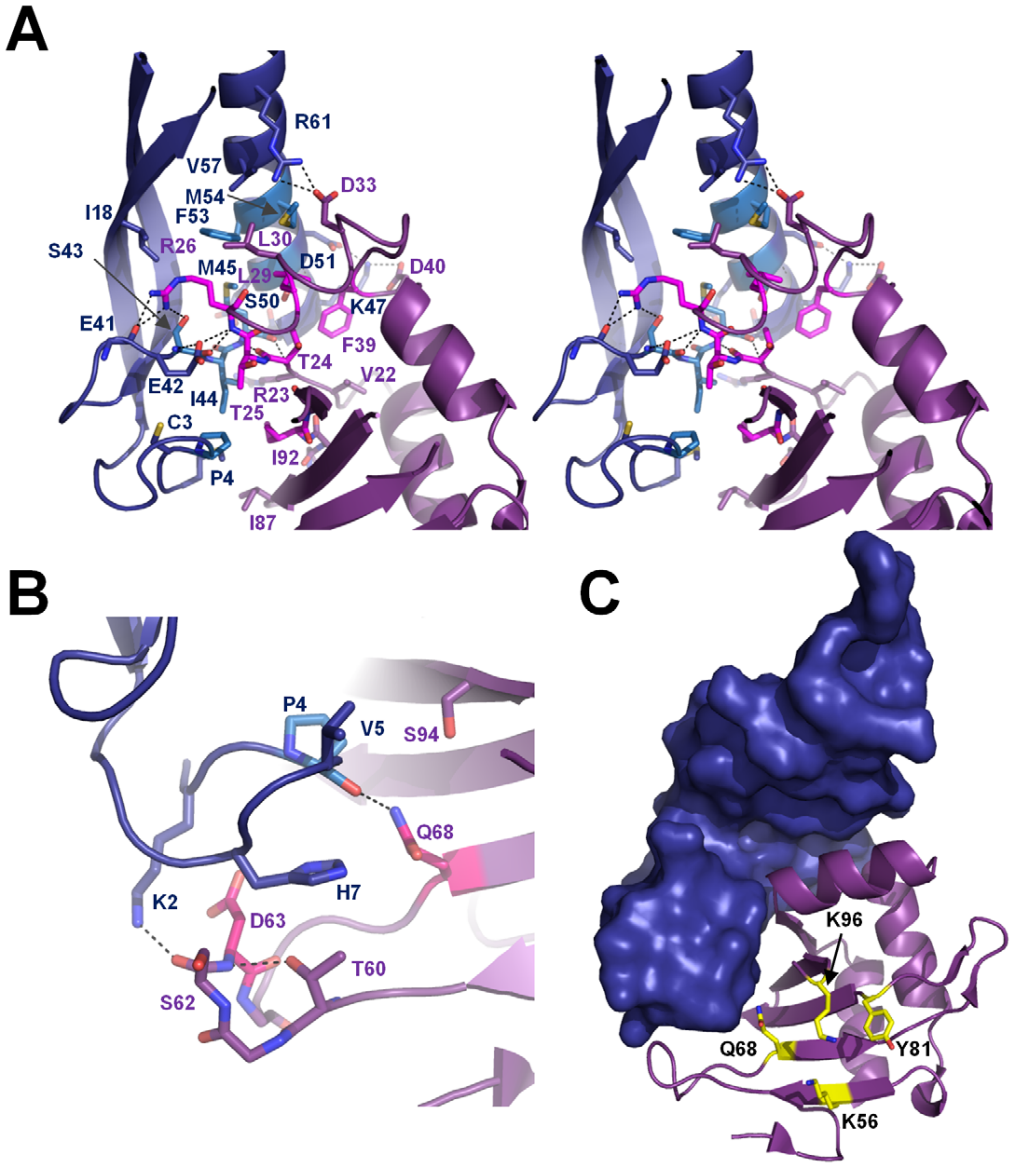
MqsA recognition of MqsR. (A) Stereoimage of the 1° interaction site between MqsR (magenta) and MqsA-N (dark blue; see also Fig. 2B), which buries 1537 Å^2^ of solvent accessible surface area (SASA). The residues that participate in the interaction are shown as sticks. Those that are completely buried upon complex formation (>80% loss of SASA) are shown in pink (MqsR) and light blue (MqsA-N). The interface is centered on MqsA-N residue M45 and is predominantly hydrophobic. Electrostatic interactions, including the hydrogen bonds formed between MqsA-N β-strand 3 (S43-M45) and MqsR β-strand 2 (V22-T25) that function to extend the MqsR β-sheet with that of MqsA-N (β1_R_-β3_R_-β4_R_-β5_R_-β6_R_-β2_R_-β3_A_-β2_A_-β1_A_), are indicated by dashed lines. (B) The 2° interaction site buries 479 Å^2^ of SASA; colors and electrostatic interactions shown as in (A). (C) The MqsR:MqsA-N complex, with MqsA-N in surface representation (dark blue) and MqsR as a ribbon diagram (magenta). The residues that play a role in MqsR-mediated toxicity (Fig. 6A) are shown as sticks in yellow. With the exception of Q68, the residues that play a role in toxicity are accessible when MqsR is bound to MqsA-N.

MqsA recognition of MqsR occurs at two distinct interfaces. The primary interface buries 1537 Å^2^ of SASA while the secondary interface buries 479 Å^2^ of SASA. A total of 2016 Å^2^ of SASA is buried upon complex formation, or 17.7% of the total SASA, well within the range expected for biological interfaces. The primary interface is centered on MqsA β-strand 3 (Ser43-Met45), which interacts with MqsR β-strand 2 (Val22-Thr25) to form a single continuous β-sheet (β1_R_-β3_R_-β4_R_-β5_R_-β6_R_-β2_R_-β3_A_-β2_A_-β1_A_; Figs. 2B, 7A) throughout the complex. Sixteen residues from MqsA-N and 12 residues from MqsR contribute to the primary interface. The interaction is hydrophobic, with Pro4, Ser43, Ile44, Met45, Ser50, Phe53, and Met54 from MqsA (light blue sticks, Fig. 7A) and Thr24, Thr25, Arg26, Leu29, Phe39 and Ile92 from MqsR (light pink sticks, Fig. 7A) becoming completely buried upon complex formation. In addition, three key electrostatic interactions are located at the periphery of the interface: (1) Arg26 from MqsR forms hydrogen bonds with the hydroxyl sidechain of Ser43 and the carbonyl of Glu41 from MqsA-N, (2) Asp33 from MqsR forms a salt bridge with Arg61 from MqsA-N and (3) Asp40 from MqsR forms a salt bridge with Lys47 from MqsA-N. The secondary interface is much smaller, being formed by four residues from MqsA-N and five residues from MqsR (Figs. 7B, S6B). The interface is centered on His7 (MqsA-N), which interacts with Thr60, Ser62, Asp63 and Gln68 from MqsR. Additional interactions are observed between Lys2, Val5 and the carbonyl of Pro4 from MqsA-N and Asp63, Gln68 and Ser94 of MqsR. MqsR residues Asp63 and Gln68 are the only residues that become buried in the secondary interface (light pink, Fig. 7B). Finally, MqsA-N does not block the MqsR active site (Fig. 7C). Instead, nearly all of the residues predicted to be important for MqsR activity are accessible in the MqsR:MqsA-N complex. This accessibility of the active site has also been observed for the RelBE system [28]. The only exception is Gln68, which is part of the secondary interface. This suggests neutralization is achieved either through cellular localization (i.e., towards DNA via MqsA-C) or through steric occlusion by blocking the interaction of MqsR with other biomolecules, such as the ribosome.

## Discussion

Our biochemical and structural analysis of the *E. coli* MqsA antitoxin and the MqsR:MqsA-N complex provide a detailed 3-dimensional structural view of the free antitoxin and the TA complex. These structures combined with our biochemical data unequivocally demonstrate that MqsR, a protein previously shown to be critical for biofilm formation [34],[35] and the most highly upregulated gene in persister cells [6] in *E. coli*, and MqsA form a novel bacterial TA system. Our data show that, as expected for a TA system, the expression of the MqsR toxin leads to growth arrest, while co-expression with its antitoxin, MqsA, rescues the growth arrest phenotype. In addition, MqsR associates with MqsA to form a tight, non-toxic complex and both MqsA alone and the MqsR:MqsA_2_:MqsR complex bind and regulate the *mqsR* promoter. Finally, the structure of MqsR reveals that it is a member of the RelE/YoeB family of bacterial RNases, which are structurally and functionally characterized bacterial toxins.

**Figure 8:**
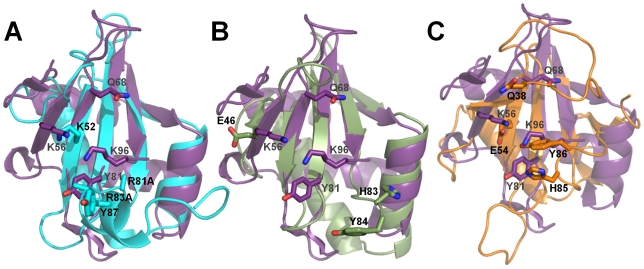
MqsR is most similar to the bacterial toxin RelE. Superposition of MqsR (magenta) with RelE (cyan; A), YoeB (green; B) and RNase Sa (orange; C) using corresponding β-strands (MqsR, β4/β5; RelE, β2/β3; YoeB, β3/β4; RNase Sa, β3/β4). Residues in RelE, YoeB and RNase SA that play a role in toxicity are shown as sticks with black labels while those in MqsR are shown as sticks with grey labels. MqsR lacks the histidine and glutamic acid residues that mediate catalysis in YoeB (E46, H83) and RNase Sa (H85, E54).

Comparison of the microbial RNase active sites between MqsR, RelE, YoeB and RNase Sa demonstrates that MqsR is most similar to RelE (Fig. 8A). In microbial RNases, such as RNase Sa, RNA binding is mediated by polar (Q38) and aromatic (Y86) residues while RNA cleavage is catalyzed by a histidine (H85) and glutamic acid (E54) residue [47],[48]. While the catalytic histidine and glutamic acid are conserved in YoeB (H83, E46), which has intrinsic endoribonuclease activity [33], they are not found in RelE, which functions as a ribosome-dependent RNase [23],[45]. These catalytic residues are also not present at the MqsR functional site (Figs. 7C, 8B, 8C). This is further supported by our demonstration that single deletion mutants of the three histidines in MqsR (H7, H64, H88; none of which overlap with the positions of H83 in YoeB and/or H85 in RNase Sa) do not attenuate MqsR-mediated toxicity. Instead, the MqsR residues that play a role in MqsR-mediated toxicity (K56, Q68, Y81 and K96) overlap best with those that are important for RelE-mediated toxicity (see K52/K56, R81/K96, R83/K96, Y87/Y81 between RelE/MqsR, respectively; Fig. 8A) [45]. This strongly suggests that MqsR, like RelE, is a ribosome-dependent RNase. This structural and functional data contradict recent results that show MqsR cleaves mRNA in the absence of the ribosome [38] and demonstrates that the precise catalytic mechanism by which mRNA is cleaved by MqsR remains to be elucidated.

However, in sharp contrast to established TA pairs such as RelE:RelB, YoeB:YefM and MazF:MazE, the MqsR:MqsA TA pair has many unique characteristics that are not observed in canonical TA systems, and thus represents the founding member of a new family of TA systems. In typical TA pairs, the antitoxin gene precedes that of the toxin. In contrast, *mqsR* precedes *mqsA*. To date, this genetic organization has only been observed in two other recently characterized TA systems, that of *higBA* [37] and *hicAB* [32]. In addition, in canonical TA systems, the toxin is larger than the antitoxin (with the exception of HicB [32]) and the toxin is basic while the antitoxin is acidic. In the MqsR:MqsA TA system, MqsA is larger than MqsR, 14.7 kD and 11.2 kD, respectively, and both proteins are basic.

Critically, one of the most significant differences between MqsR:MqsA and typical TA systems is the nature of the MqsA antitoxin itself. All other canonical antitoxins whose structures are known, including HipB [24], RelB [28], YefM [33],[49] and MazE [29], have at least one dynamic, flexible domain and all of these, with the exception of HipB, either change conformation or become ordered upon toxin binding. In contrast, MqsA is well-ordered throughout its entire sequence and its structure does not change when bound to MqsR. This explains why MqsA must be larger than MqsR; MqsA binds MqsR via a longer, folded domain, while other antitoxins bind their corresponding toxins via shorter, unstructured peptides. In addition, all other functionally characterized antitoxins bind DNA via their N-terminal domains. MqsA is the first antitoxin that has been shown experimentally to bind DNA via its C-terminal domain. The only other antitoxin predicted to bind DNA via its C- and not N-terminal domain is HicB [32],[50]. MqsA is also the first antitoxin described that requires a metal, zinc, for structural stability. Moreover, unlike most other TA inhibition mechanisms, MqsA (like its RelB homolog [28]) does not occlude the toxin active site. Finally, in addition to binding its own promoter, MqsA and the MqsR:MqsA_2_:MqsR complex also bind and regulate the promoters of genes that play roles in *E. coli* physiology, including *mcbR* and *spy*. To the best of our knowledge, this is the first time a TA system has been shown to bind the promoters of genes other than its own.

**Figure 9:**
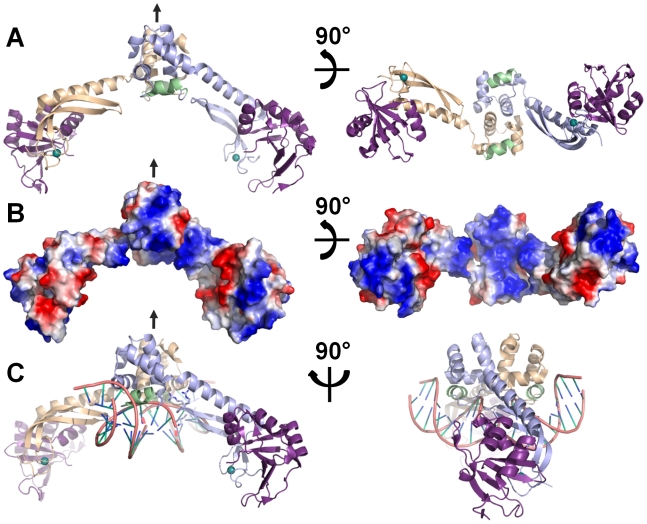
Model of the MqsR:MqsA_2_:MqsR:DNA complex. (A) The MqsA-F and MqsR:MqsA-N structures were used to generate the full MqsR:MqsA_2_:MqsR complex. The MqsA monomers are shown in light blue and light orange, with conserved MqsA-F α–helix 4 in green. MqsR is in magenta. The local two-fold symmetry axis is indicated by a black arrow. Right and left panels are rotated by 90° about the horizontal axis. (B) The electrostatic surface of the MqsR:MqsA_2_:MqsR complex (positive charges, blue; negative charges, red). The right panel is rotated about the horizontal axis, illustrating the extensive positively charged surface at the bottom of the MqsA C-terminal HTH-XRE domain. (C) Model of the MqsR:MqsA_2_:MqsR:DNA complex, which is based on the observation that all HTH-XRE DNA binding domains bind DNA using the same helix. In MqsA, this corresponds to α-helix 4. Model generation described in the methods. Because the dimerization interface differs between MqsA and the P22 C2 repressor [51], the DNA bound by MqsA must bend (illustrated by dashed lines between the two DNA segments). Two views of the MqsR:MqsA_2_:MqsR:DNA complex are shown. The left panel is the same orientation as that shown in the left panel of A. The image is rotated about the vertical axis. Very little of the MqsA-N zinc binding domain, which binds MqsR, interacts with the bound DNA. The pseudo two-fold symmetry axis is indicated by an arrow.

The MqsR:MqsA_2_:MqsR complex is oligomeric, and forms a dimer of dimers (MqsR:MqsA_2_:MqsR). Since the structure of the MqsA-N is invariant among all structures determined (MqsA-F; MqsA-N and the MqsR:MqsA-N complex), we used the MqsA-F and MqsR:MqsA-N structures to generate an accurate model of the full MqsR:MqsA_2_:MqsR complex. As can be seen in Figure 9A, the MqsR:MqsA_2_:MqsR complex forms a highly extended structure with the MqsA-C domains located at the center of the complex (MqsA monomers in light blue and light orange; α-helix 4 in green) and the MqsR toxins (magenta) bound at the periphery. In overall shape, the MqsR:MqsA_2_:MqsR complex is most similar to the MazF:MazE complex [29], which is also highly extended, although the structures of the individual proteins and oligomerization states of the complexes between the two families differ dramatically.

We and others [38] used EMSA to show that MqsA-F, MqsA-C and the MqsR:MqsA_2_:MqsR complex bind directly to the *mqsR* promoter (Figs. 1C, 1D, 4D). Therefore, MqsA likely is a transcriptional regulator of its own promoter via direct binding interactions through MqsA-C. The electrostatic surface of the MqsR:MqsA_2_:MqsR complex is shown in Figure 9B. As can be readily observed, the bottom of the MqsA-C and by direct extension, the top of the MqsA-N are nearly exclusively positively charged, typical for a strong DNA binding site. As we showed experimentally, DNA binding is localized to MqsA-C. This enabled us to exploit the similarity of the MqsA-C HTH-XRE domain to other experimentally determined HTH-XRE protein structures to generate a model for DNA binding by the MqsR:MqsA_2_:MqsR complex. All HTH-XRE DNA binding proteins whose structures have been determined in complex with DNA and which are structurally homologous to MqsA (16 protein:DNA complexes with DALI Z-scores ranging from 6.0 to 2.5), bind DNA via the same helix, the HTH-XRE DNA binding helix. In MqsA, the HTH-XRE DNA binding helix corresponds to α-helix 4 (Fig. 9A, α-helix 4 highlighted in green). Importantly, in the MqsA-F dimer, this α-helix is accessible to solvent and thus, by extension, to DNA. It is also highly positively charged, decisively contributing to the positively charged surface of the MqsA-C HTH-XRE domain dimer. Moreover, MqsA α-helix 4 is the only stretch of residues that is perfectly conserved among all MqsA proteins (Fig. S3B). Taken together, these data provide strong evidence that MqsA binds DNA via α-helix 4.

We generated a model of the MqsR:MqsA_2_:MqsR:DNA complex using the coordinates of the P22 C2 repressor bound to DNA (PDBID 2R1J) [51], the HTH-XRE protein identified to be most similar to MqsA, and the MqsR:MqsA_2_:MqsR complex (Fig. 9C). As can be seen, the DNA binds between the MqsA-N domains and makes extensive interactions with the bottom surface of the MqsA-C dimer, with α-helix 4 of both monomers projecting into the major groove of DNA. In contrast, there is minimal interaction between the DNA and MqsA-N and no interaction between the DNA and MqsR. This is perfectly consistent with the EMSA results, which show that neither MqsA-N nor MqsR:MqsA-N complex bind the *mqsR* promoter (Figs. 4C, 4E). Notably, the MqsR:MqsA_2_:MqsR:DNA complex reveals that residues at the turn connecting β-strands 1 and 2 in the MqsA N-terminal domain may interact with DNA. In support of this hypothesis, these residues, ^23^RGRK^26^, are highly basic and thus are capable of forming ionic interactions with the phosphate backbone. Thus, although MqsA-N is not capable of binding DNA alone, it may contribute to DNA binding via the interactions of these residues. This also suggests that the ability of the MqsA N- and C-terminal domains to rotate independently of one another (Fig. S5C) may help facilitate DNA binding as it would enable the MqsA-N domains to reposition themselves so residues ^23^RGRK^26^ may optimally coordinate the phosphate backbone. Since the MqsR:MqsA_2_:MqsR complex has been shown to bind DNA more tightly than MqsA alone [38], the interaction of MqsR with MqsA-N may also affect this repositioning. Finally, the model also predicts that MqsA binding induces a 30°–45° bend in the DNA. Although α-helix 4 is connected to α-helix 3 by a glycine-rich turn (^93^GGG^95^), and thus may be able to adopt a slightly different conformation when bound to DNA, it is highly unlikely that it could shift sufficiently to permit binding to unbent DNA.

Taken together, the crystal structures of full-length MqsA and the MqsR:MqsA-N complex reveal that the MqsR:MqsA TA complex is the founding member of a novel family of TA pairs. It forms a dimer of dimers, in which DNA binding and MqsR recognition by MqsA are mediated via distinct, structured domains. Because MqsR is the gene most highly upregulated in *E. coli* persister cells [6] and because it also plays an essential role in biofilm regulation and cell signaling [34],[35], these structures provide fundamental new insights into how this novel TA system participates in bacterial persistence, biofilm formation and multidrug tolerance in the *gamma*- *delta*- and *epsilon* proteobacterial classes. This work has provided the first steps for understanding this novel, unique TA system at a molecular level and provides a basis for developing novel antibacterial therapies that target TA pairs.

## Materials and Methods

### BW25113 colony formation

The bacterial strains and plasmids used in this study are listed in Table S1. Growth experiments with *E. coli* strain BW25113 were conducted in LB medium at 37°C. Growth experiments were monitored using pBS(Kan)-based plasmids [52]. To construct pBS(Kan)-based plasmids for producing MqsR, MqsA-F, and MqsR-MqsA-F from a *lac* promoter, the fragments from genomic DNA were amplified by PCR (Table S2) and directionally cloned into pBS(Kan). The toxicity of selected proteins was investigated using pBS(Kan) plasmids with 1 mM IPTG added upon inoculation. Cell viability (CFU) measured by diluting cells from 10^2^ to 10^7^ via 10-fold serial dilution steps into 0.85% NaCl solution and applying them as 10 µL drops on LB agar with kanamycin or chloramphenicol [53].

### Cloning, expression and purification

Full-length MqsA (MqsA-F, residues 1–131), the MqsA N-terminal domain (MqsA-N, residues 1–76) and the MqsA C-terminal domain (MqsA-C, residues 62–131) were subcloned into a modified pET28a vector which contained an N-terminal his_6_-tag with a TEV cleavage site [54]. Proteins were expressed in One Shot BL21 (DE3) cells (Invitrogen) and purified by sequential his_6_-tag, TEV cleavage, subtraction his_6_-tag and size-exclusion chromatography (SEC). For the MqsR:MqsA-N complex, MqsR was subcloned into the pET28a vector (Novagen) which contains an N-terminal his_6_-tag with a thrombin cleavage site. MqsA-N was subcloned into the untagged pCA21a vector (Expression Technologies, Inc.). The complex was co-expressed and purified by sequential his_6_-tag, thrombin cleavage, second subtraction his_6_-tag and SEC.

### Crystallization

Crystals of MqsA-F were obtained using the sitting drop vapor diffusion method at 4°C. Crystals were grown by mixing 0.2 µL of MqsA-F protein (3.5 mg/ml; 10 mM Tris pH 7.0, 50 mM NaCl, 0.5 mM TCEP, 5% (v/v) ethanol) with 0.2 µL of precipitant (75 mM Bis-Tris, pH 5.5, 150 mM MgCl_2_, and 19% (w/v) PEG3350). Crystals of MqsR:MqsA-N were obtained using the sitting drop vapor diffusion method at 4°C. Crystals were grown by mixing 0.2 µL MqsR:MqsA-N protein complex (14 mg/ml; 10 mM Tris pH 7.5, 100 mM NaCl, 0.5 mM TCEP) with 0.4 µL of precipitant (0.1 M Bis-Tris pH 5.5 and 25% (w/v) PEG3350). MqsA-F and MqsR:MqsA-N crystals were cryoprotected in precipitant containing 20% glycerol and frozen in liquid nitrogen for data collection.

### Data collection and processing

Data for both MqsA-F and the MqsR:MqsA-N complex were collected at the National Synchrotron Light Source, beamline X6A at Brookhaven National Laboratory. Native data for MqsA-F was collected from a single crystal at a single wavelength. Anomalous data for the MqsR:MqsA-N complex was collected from a single crystal; a three-wavelength MAD experiment was performed collecting data at the experimentally determined Zn/K absorption edge, the inflection point and a high energy remote wavelength. Data were processed and scaled using HKL2000 [55].

### Model building and refinement

*MqsA-F*: Molecular replacement (Phaser [56]) using the structures of the MqsA individual domains (MqsA-N and MqsA-C; structure determination of MqsA-N and MqsA-C described in Protocol S1, Figs. S5A, S5B and Tables S3, S4) as search models resulted in a single solution, with two molecules of each domain present in the asymmetric unit. The resulting electron density maps were readily interpretable. Model building was performed in Coot [57] followed by restrained refinement in REFMAC 5.2.0019 [58]. As observed for the structure of MqsA C-terminal domain alone (Fig. S3B), Gln108 is methylated at N5. *MqsR:MqsA-N complex*: SOLVE was used to determine the positions of the two expected zinc atoms. Initial phases to 2.0 Å were improved with density modification in RESOLVE. An initial model built by ARP/wARP served as the basis for subsequent manual model building and refinement. Iterative cycles of refinement in REFMAC against the high energy remote data were used to complete and refine the model. Data collection, model and refinement statistics for both structures are reported in Table 1. Structure validation and stereochemistry analysis was performed with Molprobity [59] and SFCHECK [60].

### Electrophoretic mobility shift assays (EMSA)

The targeted promoter regions (150–250 bp upstream of the start codon using primers reported in Table S2) were amplified, purified and labeled with biotin using the Biotin 3′-end DNA Labeling Kit (Pierce Biotechnology). After binding the proteins (200 ng; MqsA-F, MqsA-N, MqsA-C, MqsR:MqsA-N and MqsR:MqsA-F) with biotin-labeled target promoters (5 ng), electrophoresis was conducted at 100 V at 4°C using a 6% DNA retardation gel (Invitrogen). The binding mixtures were transferred to a nylon membrane (Roche Diagnostics GmbH) using a Mini Trans-Blot Electrophoretic Transfer Cell (Bio-Rad), and 3′-biotin-labeled DNA was detected with the Light-Shift Chemiluminescent EMSA kit (Pierce Biotechnology).

### MqsR toxicity assays

Mutagenesis of MqsR was carried out using the Quikchange mutagenesis kit (Stratagene) and sequence verified. Growth experiments were conducted in LB at 18°C and all mutants were tested in parallel. Briefly, 10 ml of an overnight culture was used to inoculate 1 L of LB and then incubated at 37°C with rigorous shaking. Once a mutant culture reached an OD_600_ of 0.4, it was cooled on ice. After all the mutants reached an OD_600_ of 0.4, the cultures were cooled for an additional hour, induced with 0.5 mM IPTG and incubated at 18°C with vigorous shaking. OD_600_ measurements were taken every hour for 20 hours. Growth assays for MqsR:MqsA-N and MqsR:MqsA-C were carried out similarly, except expression was induced at an OD_600_ of 0.7.

### MqsR:MqsA_2_:MqsR:DNA complex modeling

The P22 C2 repressor protein (PDBID 2R1J) [51] was identified as the closest structural homolog to the MqsA C-terminal domain (DALI Z-score  = 6.0), whose DNA-bound structure was known. The MqsA:DNA complex was modeled by superimposing the conserved HTH-XRE helices from both proteins (MqsA residues 98–105; P22R residues 34–41) and mapping the rotated DNA coordinates onto one MqsA monomer. This MqsA monomer:DNA complex was then superimposed onto the symmetry-related MqsA monomer to obtain the rotated DNA coordinates for the second monomer.

### Genes, proteins and structures discussed in the text

The genes/proteins mentioned in the text include (UniProtKB/Swiss-prot ID unless stated otherwise): MqsR/YgiU (Q46865), MqsA/YgiT (Q46864), McbR (P76114), Spy (P77754), RelE (P0C077), RelB (P0C079), MazE (P0AE72), MazF (P0AE70), DinJ (Q8X7Q6), YaFQ (Q47149), HipA (P23874), HipB (P23873), HicA (P76106), HicB (P67697), YefM (P69346), YoeB (P69348), HigA (Q9KMG4/Q9KMA5), HigB (Q9KMG5/Q9KMA6). The PDB files mentioned in the text are RelE (PDBID 2KC8), YoeB (PDBID 2A6Q) and RNase Sa (PDBID 1RSN).

### Coordinates

The structure factors and coordinates for MqsA-F and the MqsR:MqsA-N complex have been deposited with the Protein Databank with accession numbers 3GN5 and 3HI2, respectively.

## Supporting Information

Figure S1 MqsR and MqsA are a Toxin:Antitoxin Pair. The effect of MqsR, MqsA-F and the MqsR:MqsA-F complex on cell growth (A, D), cell viability (CFU/ml) (B, E) and colony formation (C) for *E. coli* strain MG1655 (A–C) and BW25113 (D, E) containing pBS(Kan) (black circle, empty plasmid), pBS(Kan)-*mqsA-F* (open circle), pBS(Kan)-*mqsR* (black triangle) and pBS(Kan)-*mqsR-mqsA-F* (open triangle) at 37°C in LB with 1 mM IPTG induction upon inoculation (incubation time  = 0 min). Electrophoretic mobility shift assay controls that show both MqsR:MqsA-F (F) and MqsA-F (G) do not bind the P*tomB* DNA probe, which was used as a non-specific competitive control for the EMSA assays shown in Figure 1.
(1.25 MB TIF)

Figure S2 MqsA is Susceptible to Proteolytic Degradation. SDS-PAGE analysis of MqsA-F incubated with chymotrypsin, trypsin or proteinase K for 5, 15, 30 and 60 minutes at 30°C. The upper band indicated with an arrow corresponds to undigested MqsA_1–131_ (14.9 kDa), while the proteolytic fragments are denoted with *. The trypsin digested samples analyzed by MALDI-TOF MS and LC-MS/MS are boxed. Proteolytic digestion of a stable, folded domain (SPAR PDZ domain) and an intrinsically unstructured protein (DARPP-32) are shown on the right for comparison.
(0.82 MB TIF)

Figure S3 MqsR and MqsA are conserved among multiple bacterial species. Sequence alignment of MqsR (A) and MqsA (B). Secondary structural elements for MqsR and MqsA are represented as cylinders (α-helices) or arrows (β-strands) and shaded according to the protein/domain to which they belong (MqsR, magenta; MqsA-N, MqsA N-terminal zinc binding domain, dark blue; MqsA-C, MqsA C-terminal HTH-XRE domain, light blue, with the exception of the HTH-XRE conserved helix, which is green). Identical residues are highlighted in cyan whereas similar residues are shaded in gray; symbols below the sequence: ‘*’ identical residues; ‘:’ highly similar residues; ‘.’ similar residues.
(1.59 MB TIF)

Figure S4 Constructs used in this study. MqsA residues 62–76 represent the ‘linker’ between the two domains of MqsA. Subsequent structure determination demonstrates that the physical linker between the MqsA N- and C-terminal domains is short, centered on residue T68.
(0.20 MB TIF)

Figure S5 Crystal Structures of the Individual MqsA Domains and their Independent Rotation in the MqsA dimer.(A) Ribbon model of MqsA-N. Bound zinc is illustrated as a teal sphere. (B) Ribbon model of MqsA-C. α-helix 4, which is predicted to mediate DNA binding, is shaded in green. (C) The N- and C-terminal domains of MqsA rotate independently via the short flexible linker. Both monomers of MqsA were superimposed on the MqsA-C domains (light orange, light blue) to illustrate the relative rotation of the MqsA-N domains (dark orange, dark blue), resulting in a shift of the bound zinc ions by 18 Å.
(1.46 MB TIF)

Figure S6 Stereoimages of MqsA and MqsR. (A) Stick representation of the MqsA zinc binding pocket (residues K2-M11 and C37-E42) with σA-weighted 2m*F_o_*-d*F_c_* map at 1.5 σ. MqsA in green, zinc ion in teal. (B) Stick representation of a portion of the MqsR:MqsA secondary interface (MqsR residues T59-Q68 in blue and MqsA residues K2-H7 in green) with σA-weighted 2m*F_o_*-d*F_c_* map at 1.5 σ.
(2.08 MB TIF)

Figure S7 Semi-log plots of MqsR toxicity assays illustrated in Figures 4A and 6A. (A) Semi-log plot of the growth curves of the co-expression of MqsR with the N-terminal domain of MqsA (MqsR:MqsA-N, grey square) and the C-terminal domain of MqsA (MqsR:MqsA-C, black diamond) in BL21 (DE3) cells. Induction of protein expression using 0.5 mM IPTG corresponds to t = 0 (arrow; expression carried out at 18°C, following the same protocol used to produce the proteins for structural studies). Co-expression of MqsR with MqsA-C leads to growth arrest while co-expression with MqsA-N results in robust growth. (B) Semi-log plot of the growth curves of cells over-expressing WT MqsR and seven MqsR mutants. MqsR mutants K56A, Q68A, Y81A and K96A show decreased toxicity compared to WT MqsR, as evidenced by their ability to grow following induction with 0.5 mM IPTG at t = 0 (arrow; expression carried out at 18°C, following the same protocol used to produce the proteins for structural studies). In contrast, induction of MqsR mutants Y55A, M58A and R72A, like WT, lead to growth arrest.
(0.39 MB TIF)

Protocol S1 Supporting Protocols and References.
(0.07 MB PDF)

Table S1 Bacterial strains and plasmids used in this study.
(0.05 MB PDF)

Table S2 Oligonucleotides used for this study.
(0.02 MB PDF)

Table S3 Data collection and refinement statistics for MqsA-N.
(0.05 MB PDF)

Table S4 Data collection and refinement statistics for MqsA-C.
(0.05 MB PDF)
