# Supplementary figures and images for: Three Dimensional Structure of the MqsR:MqsA Complex: A Novel TA Pair Comprised of a Toxin Homologous to RelE and an Antitoxin with Unique Properties
Source: PLoS Pathog. 2009 Dec 24;5(12):e1000706. doi: 10.1371/journal.ppat.1000706 (PMC2791442; doi:10.1371/journal.ppat.1000706)

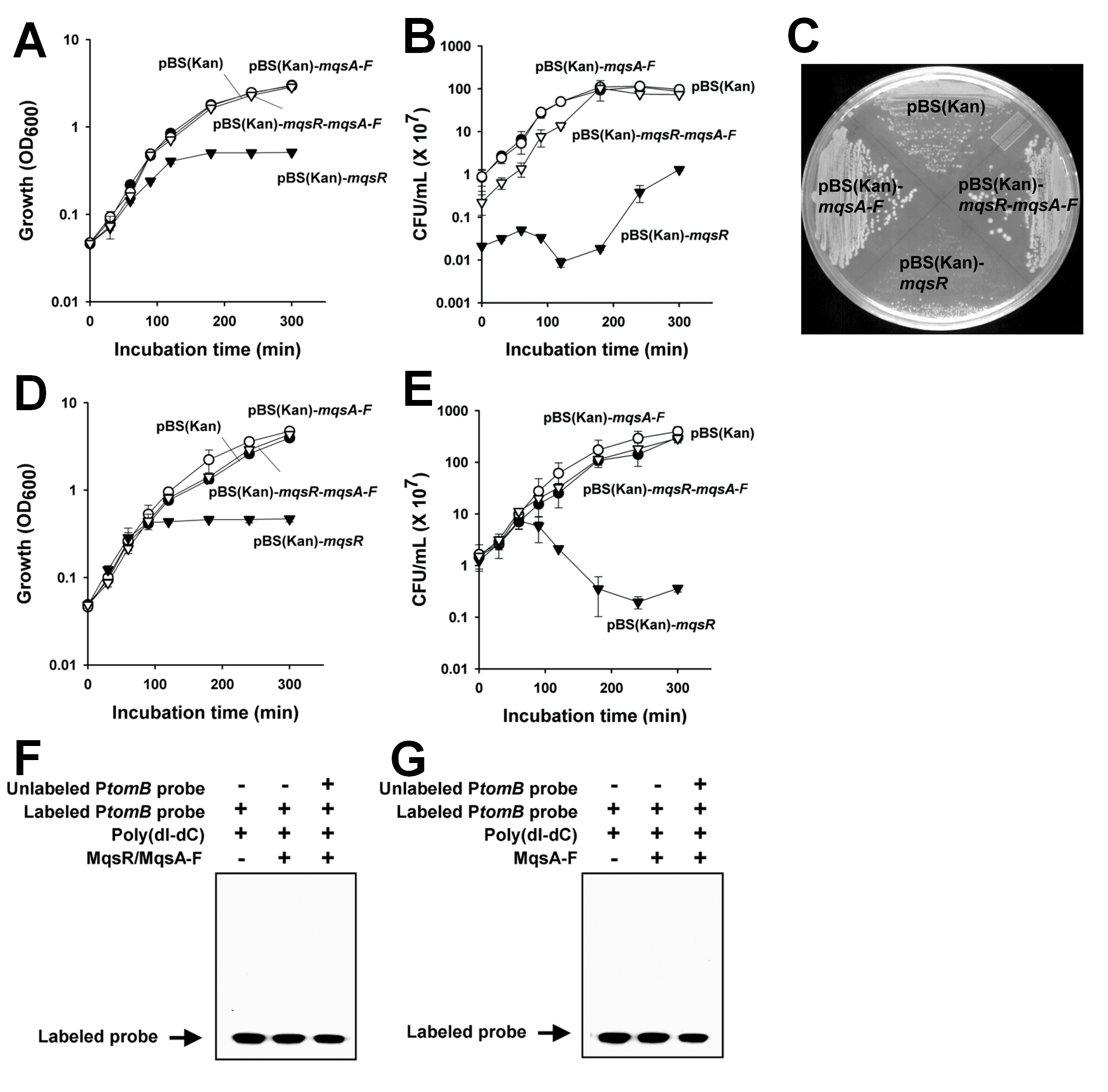

Supplement: Figure S1 — MqsR and MqsA are a Toxin:Antitoxin Pair. The effect of MqsR, MqsA-F and the MqsR:MqsA-F complex on cell growth (A, D), cell viability (CFU/ml) (B, E) and colony formation (C) for E. coli strain MG1655 (A–C) and BW25113 (D, E) containing pBS(Kan) (black circle, empty plasmid), pBS(Kan)-mqsA-F (open circle), pBS(Kan)-mqsR (black triangle) and pBS(Kan)-mqsR-mqsA-F (open triangle) at 37°C in LB with 1 mM IPTG induction upon inoculation (incubation time = 0 min). Electrophoretic mobility shift assay controls that show both MqsR:MqsA-F (F) and MqsA-F (G) do not bind the PtomB DNA probe, which was used as a non-specific competitive control for the EMSA assays shown in Figure 1. (1.25 MB TIF) [file ppat.1000706.s001.tif]

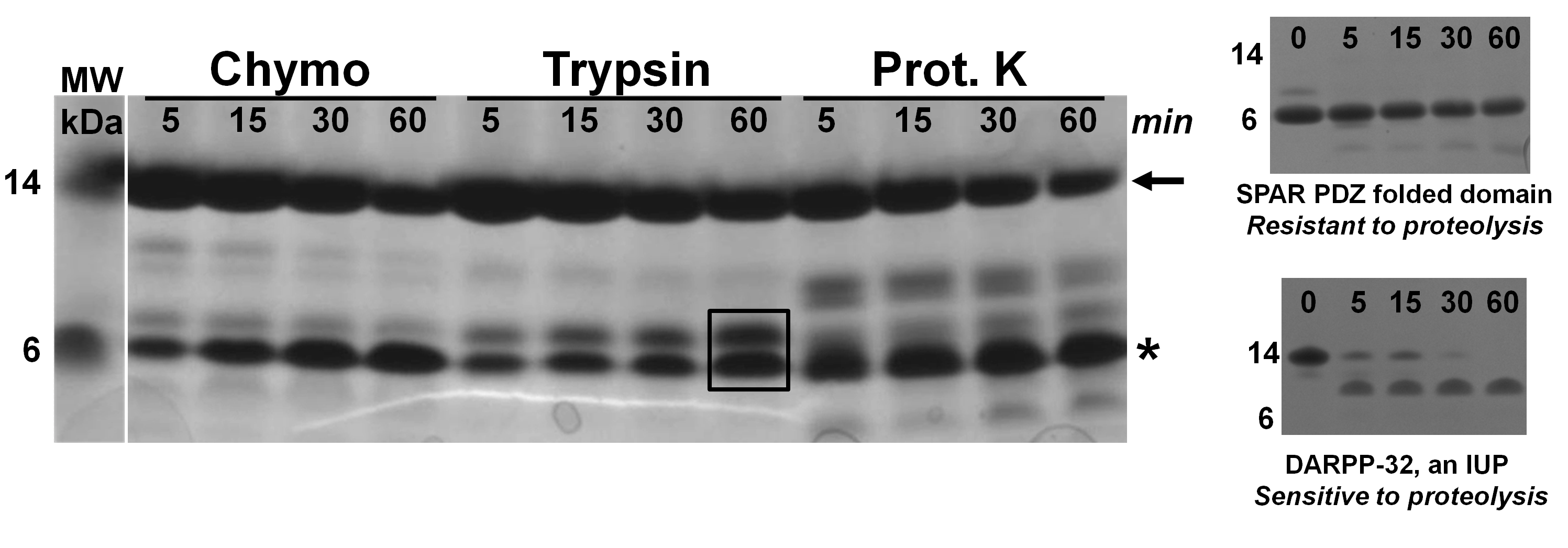

Supplement: Figure S2 — MqsA is Susceptible to Proteolytic Degradation. SDS-PAGE analysis of MqsA-F incubated with chymotrypsin, trypsin or proteinase K for 5, 15, 30 and 60 minutes at 30°C. The upper band indicated with an arrow corresponds to undigested MqsA1–131 (14.9 kDa), while the proteolytic fragments are denoted with *. The trypsin digested samples analyzed by MALDI-TOF MS and LC-MS/MS are boxed. Proteolytic digestion of a stable, folded domain (SPAR PDZ domain) and an intrinsically unstructured protein (DARPP-32) are shown on the right for comparison. (0.82 MB TIF) [file ppat.1000706.s002.tif]

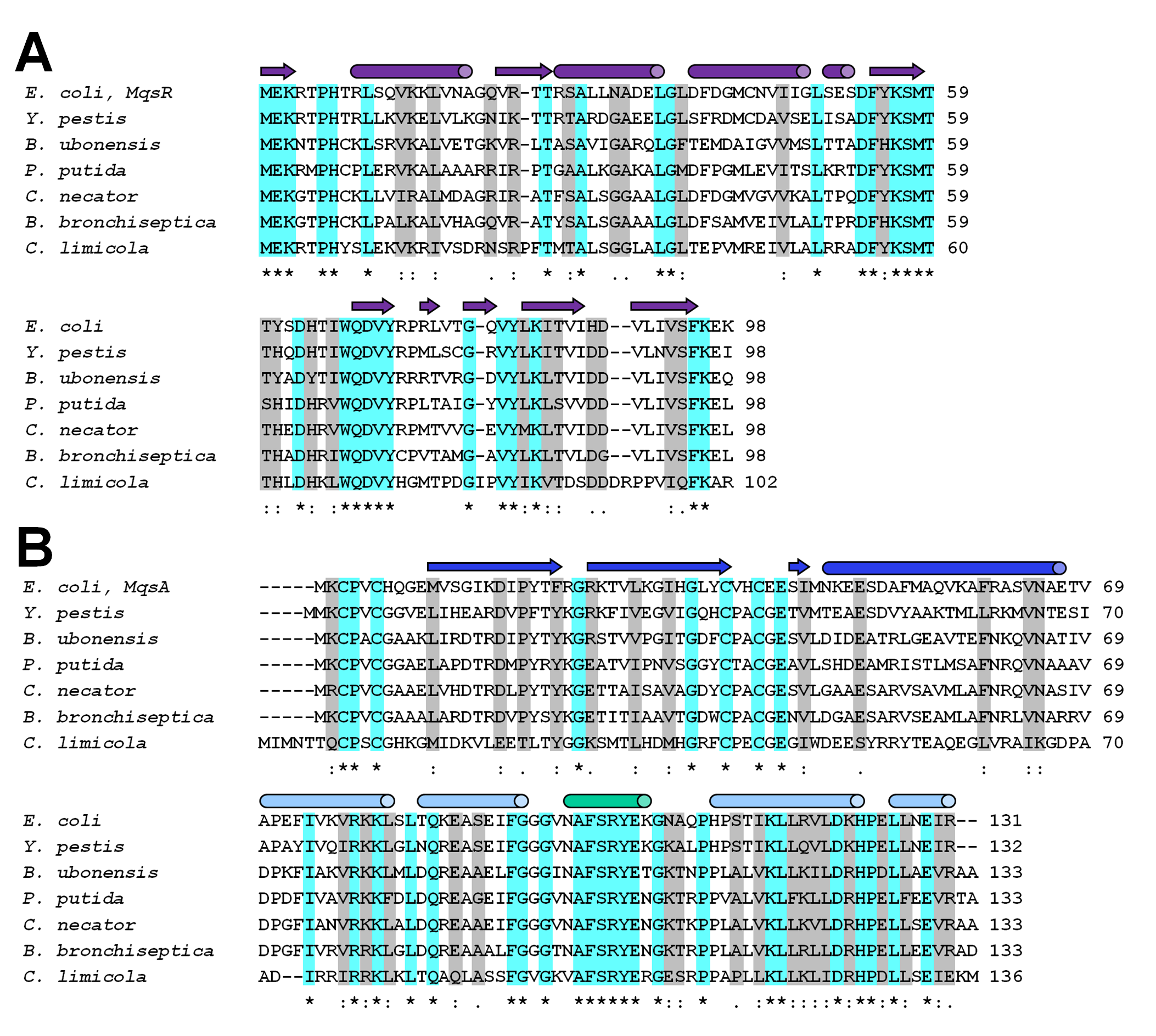

Supplement: Figure S3 — MqsR and MqsA are conserved among multiple bacterial species. Sequence alignment of MqsR (A) and MqsA (B). Secondary structural elements for MqsR and MqsA are represented as cylinders (α-helices) or arrows (β-strands) and shaded according to the protein/domain to which they belong (MqsR, magenta; MqsA-N, MqsA N-terminal zinc binding domain, dark blue; MqsA-C, MqsA C-terminal HTH-XRE domain, light blue, with the exception of the HTH-XRE conserved helix, which is green). Identical residues are highlighted in cyan whereas similar residues are shaded in gray; symbols below the sequence: ‘*’ identical residues; ‘:’ highly similar residues; ‘.’ similar residues. (1.59 MB TIF) [file ppat.1000706.s003.tif]

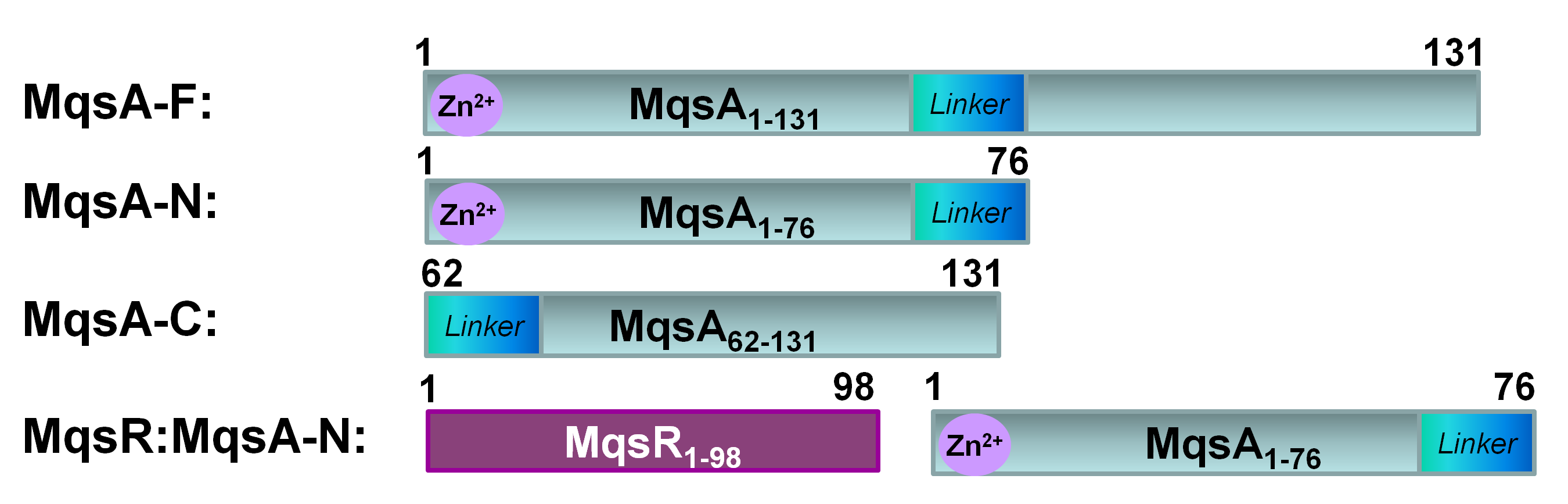

Supplement: Figure S4 — Constructs used in this study. MqsA residues 62–76 represent the ‘linker’ between the two domains of MqsA. Subsequent structure determination demonstrates that the physical linker between the MqsA N- and C-terminal domains is short, centered on residue T68. (0.20 MB TIF) [file ppat.1000706.s004.tif]

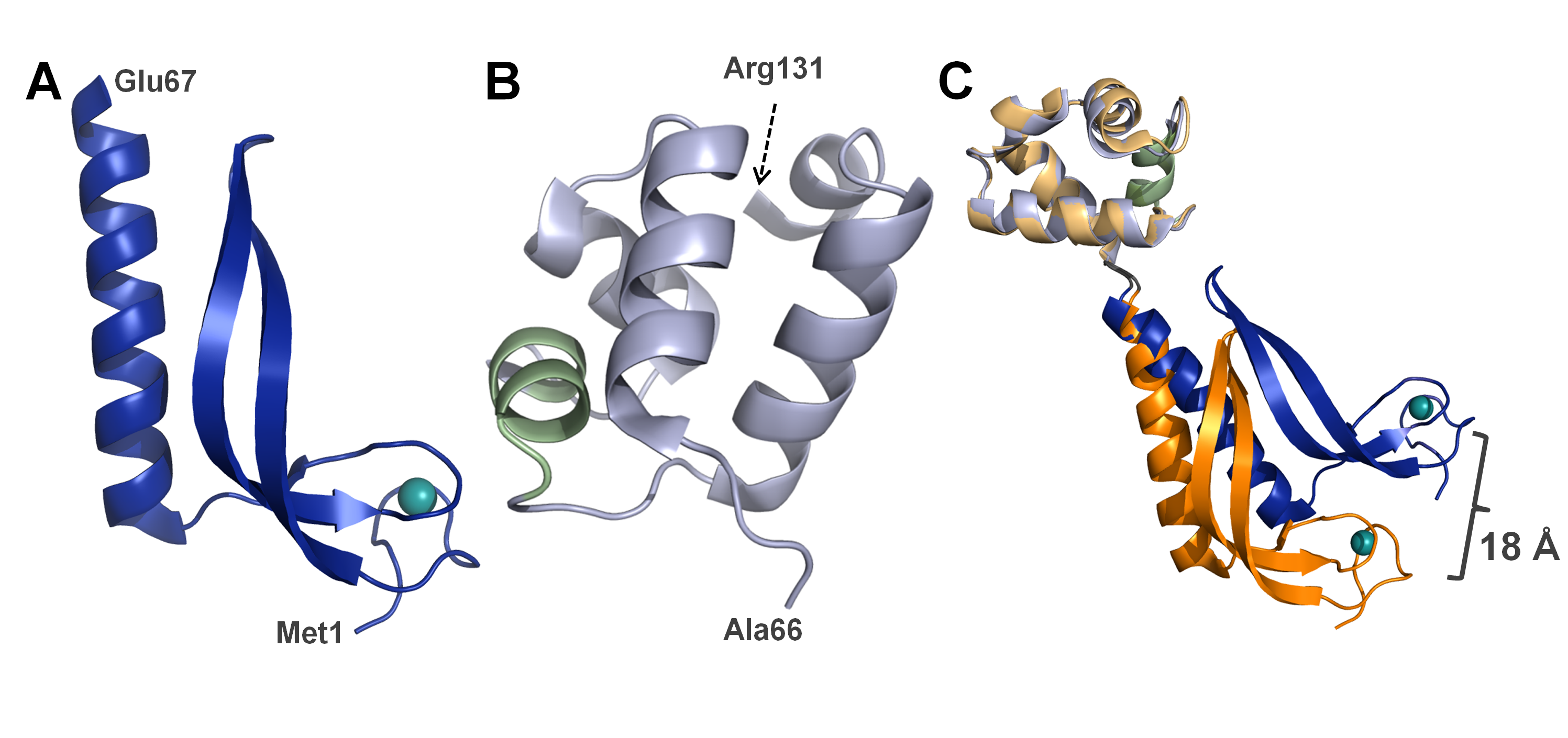

Supplement: Figure S5 — Crystal Structures of the Individual MqsA Domains and their Independent Rotation in the MqsA dimer.(A) Ribbon model of MqsA-N. Bound zinc is illustrated as a teal sphere. (B) Ribbon model of MqsA-C. α-helix 4, which is predicted to mediate DNA binding, is shaded in green. (C) The N- and C-terminal domains of MqsA rotate independently via the short flexible linker. Both monomers of MqsA were superimposed on the MqsA-C domains (light orange, light blue) to illustrate the relative rotation of the MqsA-N domains (dark orange, dark blue), resulting in a shift of the bound zinc ions by 18 Å. (1.46 MB TIF) [file ppat.1000706.s005.tif]

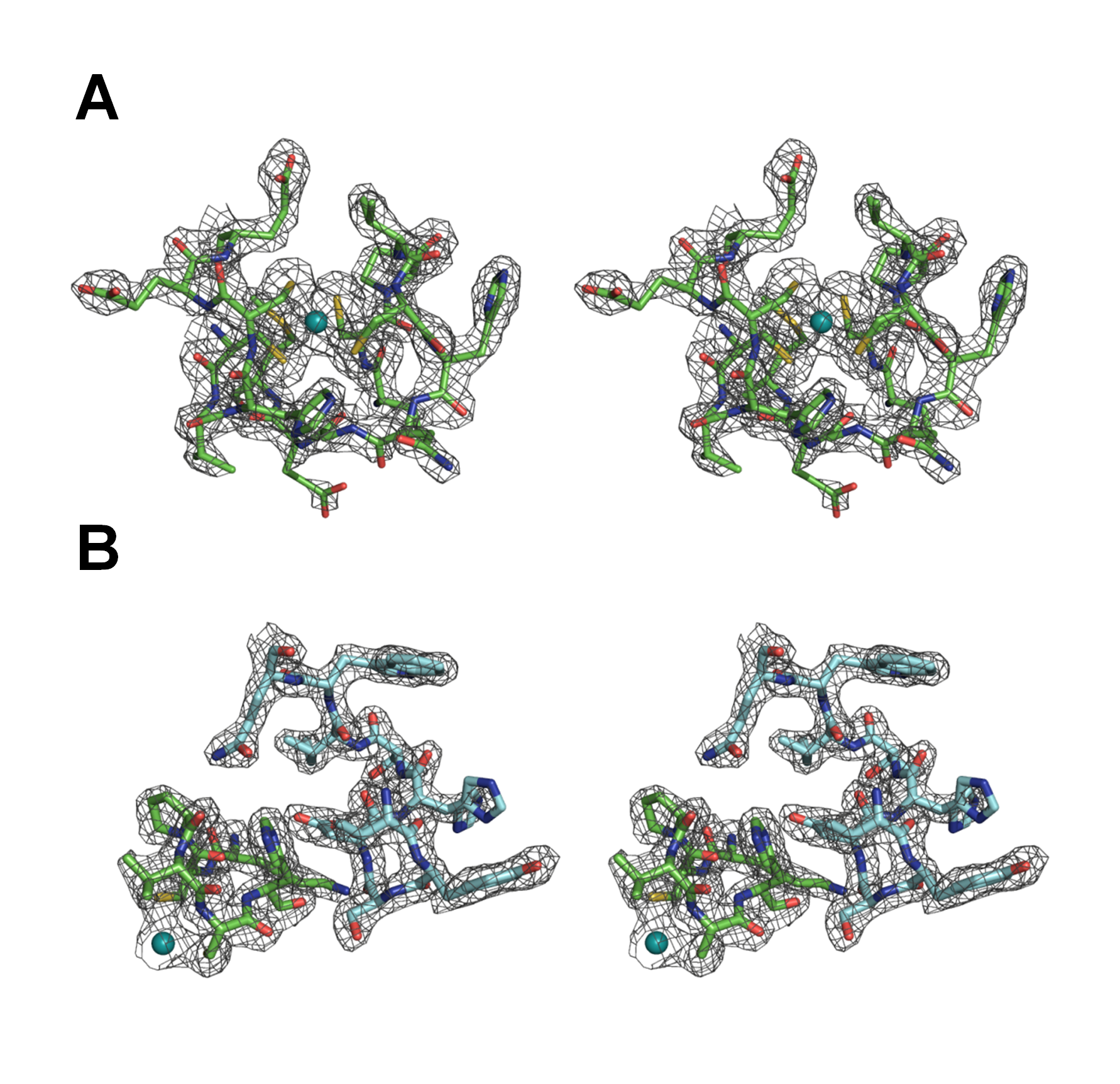

Supplement: Figure S6 — Stereoimages of MqsA and MqsR. (A) Stick representation of the MqsA zinc binding pocket (residues K2-M11 and C37-E42) with σA-weighted 2mFo-dFc map at 1.5 σ. MqsA in green, zinc ion in teal. (B) Stick representation of a portion of the MqsR:MqsA secondary interface (MqsR residues T59-Q68 in blue and MqsA residues K2-H7 in green) with σA-weighted 2mFo-dFc map at 1.5 σ. (2.08 MB TIF) [file ppat.1000706.s006.tif]

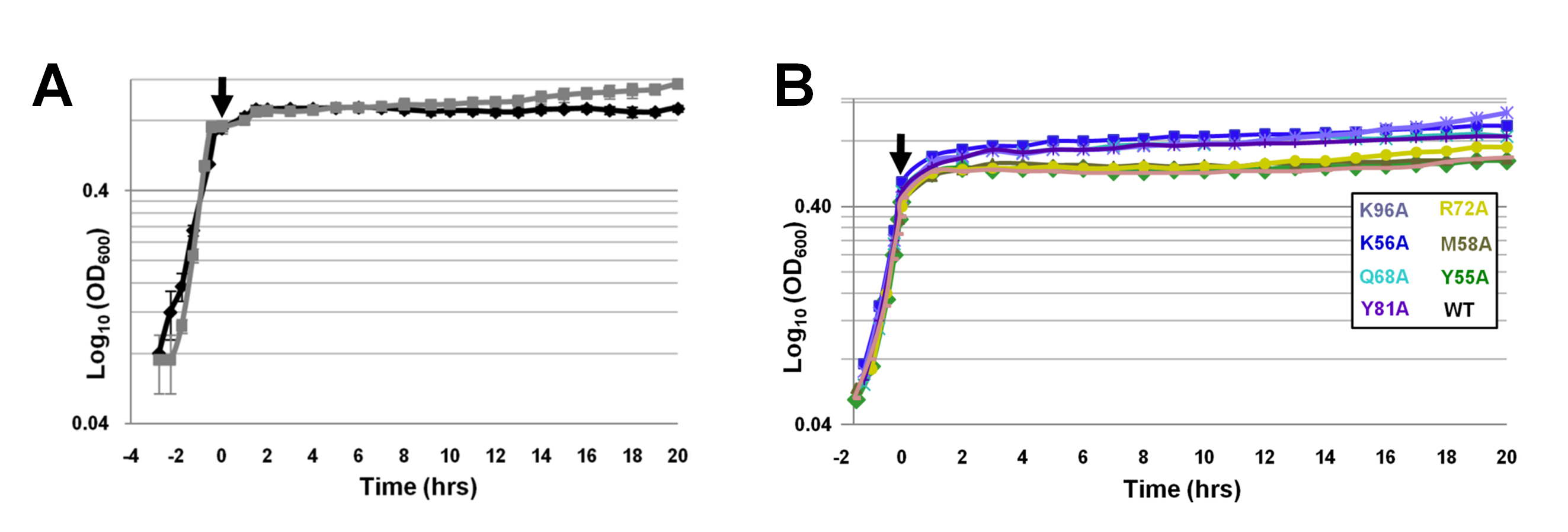

Supplement: Figure S7 — Semi-log plots of MqsR toxicity assays illustrated in Figures 4A and 6A. (A) Semi-log plot of the growth curves of the co-expression of MqsR with the N-terminal domain of MqsA (MqsR:MqsA-N, grey square) and the C-terminal domain of MqsA (MqsR:MqsA-C, black diamond) in BL21 (DE3) cells. Induction of protein expression using 0.5 mM IPTG corresponds to t = 0 (arrow; expression carried out at 18°C, following the same protocol used to produce the proteins for structural studies). Co-expression of MqsR with MqsA-C leads to growth arrest while co-expression with MqsA-N results in robust growth. (B) Semi-log plot of the growth curves of cells over-expressing WT MqsR and seven MqsR mutants. MqsR mutants K56A, Q68A, Y81A and K96A show decreased toxicity compared to WT MqsR, as evidenced by their ability to grow following induction with 0.5 mM IPTG at t = 0 (arrow; expression carried out at 18°C, following the same protocol used to produce the proteins for structural studies). In contrast, induction of MqsR mutants Y55A, M58A and R72A, like WT, lead to growth arrest. (0.39 MB TIF) [file ppat.1000706.s007.tif]
