# Supplementary material for: Three Dimensional Structure of the MqsR:MqsA Complex: A Novel TA Pair Comprised of a Toxin Homologous to RelE and an Antitoxin with Unique Properties
Source: PLoS Pathog. 2009 Dec 24;5(12):e1000706. doi: 10.1371/journal.ppat.1000706 (PMC2791442; doi:10.1371/journal.ppat.1000706)
Supplement: Protocol S1 — Supporting Protocols and References. (0.07 MB PDF) [file ppat.1000706.s008.pdf]

## Supporting Protocols and References

## Three Dimensional Structure of the MqsR:MqsA Complex: a Novel TA Pair comprised of a Toxin Homologous to RelE and an Antitoxin with Unique Properties

Breann L. Brown[#], Simina Grigoriu[#], Younghoon Kim, Jennifer M. Arruda, Andrew Davenport, Thomas K. Wood, Wolfgang Peti and Rebecca Page

## Supporting Protocols

### BW25113/MG1655 growth assays

The bacterial strains and plasmids used in this study are listed in Table S1. Growth experiments with *E. coli* strain BW25113 and MG1655 were conducted in LB medium at 37°C. Growth experiments were monitored using pBS(Kan)-based plasmids [1]. To construct pBS(Kan)-based plasmids for producing MqsR, MqsA-F, and MqsR-MqsA-F from a *lac* promoter, the fragments from genomic DNA were amplified by PCR (Table S2) and directionally cloned into pBS(Kan). The toxicity of selected proteins was investigated using pBS(Kan) plasmids with 1 mM IPTG added upon inoculation. Growth measured using culture optical density at 600 nm ($OD_{600}$). Cell viability (CFU) measured by diluting cells from $10^2$ to $10^7$ via 10-fold serial dilution steps into 0.85% NaCl solution and applying them as 10 µL drops on LB agar with kanamycin or chloramphenicol [2]. Two independent cultures were used for each strain. Data shown (Figs. 1, S1) represents the average of the two measurements with the standard deviation shown as error bars.

### Proteolytic Digestion

MqsA-F was incubated at 30°C with trypsin, chymostrypsin, or proteinase K at a 1000:1 ratio (100 µg of protein:100 ng protease) for 5, 15, 30, or 60 min. The digestion reactions were stopped by heating the samples at 100°C and cleavage was identified using SDS-PAGE analysis. Proteolytic digestion of a stable, folded domain (SPAR PDZ domain [3]) and an intrinsically unstructured protein (DARPP-32 [4]) were carried out similarly. Cleavage products at 6 and 7 kDa were excised from the SDS-PAGE gel, prepared according to standard protocols and analyzed using LC-MS/MS (LTQ mass spectrometer; Ottawa Institute of Systems Biology Proteomics Facility) coupled with data analysis using Mascot.

### Mutagenesis of MqsA-C

Mutagenesis of MqsA-C L81M was carried out using the Quikchange Mutagenesis Kit (Stratagene) using manufacture protocols. Protein expression in selenomethionine medium was carried out as previously described [5].

### Crystallization of MqsA-N (residues 1-76) and MqsA-C (residues 62-131)

*MqsA-N*: Crystals were obtained using two sequential rounds of seeding. Initially, MqsA-N formed thin needle-like crystals in 0.1 M CHES pH 9.5, 20% (w/v) PEG8000 using the sitting drop vapor diffusion method. These crystals were crushed, diluted and used as crystallization microseed solution (seed bead; Hampton Research) for subsequent crystallization trials. Crystals were obtained in a new condition, which contained 0.1 M Tris pH 8.5, 0.2 M $Li_2SO_4$, 30% (w/v) PEG4000 (0.3 µL protein, 0.2 µL crystallization condition, and 0.1 µL seed solution; sitting drop vapor diffusion). These crystals, which also formed thin needles, were then crushed, diluted and used as crystallization microseed solution for a second round of seeding which produced the MqsA-N crystals used for data collection. Final crystals were grown in 0.1 M Tris pH 8.1, 0.2 M $Na_2SeO_4$, 30% (w/v) PEG4000 using the microbatch method at 22°C. Crystals were cryoprotected by a 30 second soak in mother liquor supplemented with 25% glycerol and immediately flash frozen in liquid $N_2$. *MqsA-C*: Crystals were obtained at 4°C in 0.1 M Tris pH 8.5, 5% (w/v) PEG8000, 10% (v/v) glycerol, 20% (v/v) PEG300 using sitting drop vapor diffusion with 2:1 v/v ratio of protein to crystallization condition. MqsA-C L81M SeMet-derivative protein crystallized in 0.1 M HEPES pH 7.5, 0.2 M NaCl, 40% (v/v) PEG300 at 4°C. Both crystallization conditions for native and SeMet crystals were sufficient for cryoprotection and crystals were frozen by direct transfer to liquid nitrogen.

### Data Collection and Structure Determination of MqsA-N and MqsA-C

All data were collected at the National Synchrotron Light Source Beamline X6A at 100 K using an ADSC Q270 (MqsA-N) or Q210 (MqsA-C) CCD detector and processed using HKL2000 [6]. *MqsA-N*: The crystal structure of MqsA-N was solved using single wavelength anomalous dispersion (SAD). Experimental phases for MqsA-N were determined using HKL2MAP with SHELXC/D/E [7]. One heavy atom was identified, which, after automated model building with the program ARP/wARP [8,9], was determined to be a metal coordinated by the four N-terminal domain cysteines. X-ray wavelength scans at the K-$\alpha_1$ absorption edges of zinc, nickel and cobalt confirmed that the coordinated metal was zinc. MqsA-N was refined against the data collected at the high energy remote wavelength. The model was completed with iterative rounds of model building using Coot [10] followed by restrained refinement in REFMAC 5.2.0019 [11] with a TLS model determined using the TLSMD server [12]. Residues and sidechains for which no density was observed were not modeled. Data collection, model and refinement statistics are reported in Table S3 and the structure is illustrated in Figure S5A. The final MqsA-N structure was refined to 1.7 Å with an R-factor of 16.5%

($R_{free}$ = 18.6%) and contains MqsA residues 1-67, one zinc ion, and 97 water molecules; residues 68-76 were not observed in the electron density. The Matthews coefficient ($V_m$) for MqsA-N is 2.48 $Å^3$/Da and the estimated solvent content is 50.4%. One molecule of MqsA-N was present in the asymmetric unit. Analysis of the crystal contacts was carried out using AREAIMOL (which measures the buried solvent accessible surface area) [13] and SC (which measures the surface complementarity of protein:protein interfaces) [14], and confirms that the oligomerization state of MqsA-N is monomeric. Structure validation and stereochemistry analysis was performed with Molprobity [15] and SFCHECK [16].

*MqsA-C*: The structure of MqsA-C was solved using the multi-wavelength anomalous dispersion (MAD) method. Data collected at the peak, inflection, and remote wavelengths were indexed, integrated, and scaled using HKL2000 [6]. Experimental phases were determined using SOLVE/RESOLVE [17]. Automated model building was performed with ARP/wARP [8,9] with 10 cycles of autobuilding and REFMAC [11] refinement. The model was refined against native data. The model was completed with iterative rounds of model building using Coot [10] followed by anisotropic restrained refinement in REFMAC 5.2.0019 [11]. Residues and sidechains for which no density was observed were not modeled. Data collection, model and refinement statistics are reported in Table S4 and the structure is illustrated in Figure S5B. The final structure of MqsA-C was refined to 1.4 Å with an R-factor of 15.5% ($R_{free}$ = 18.2%). The model includes MqsA residues 66-131 and 80 water molecules; residues 62-65 were not observed in the electron density map. Strong difference density (>7σ) was observed suggesting Gln108 is methylated, which we confirmed using LC-MS/MS (Thermo LTQ MS; Brown University Proteomics facility). The Matthews coefficient ($V_m$) for MqsA-C is 2.18 $Å^3$/Da and the estimated solvent content is 43.5%. One molecule of MqsA-C was present in the asymmetric unit. Analysis of the crystal contacts using AREAIMOL [13] and SC [14] confirmed that the oligomerization state of MqsA-C is dimeric, with the monomers of the dimer related by crystallographic 2-fold symmetry. Structure validation and stereochemistry analysis was performed with Molprobity [15] and SFCHECK [16].

### Coordinates

The structure factors and coordinates for MqsA-N and MqsA-C have been deposited with the Protein Databank with accession numbers 3GA8 and 3FMY, respectively.

### Supporting References

1. Canada KA, Iwashita S, Shim H, Wood TK (2002) Directed evolution of toluene ortho-monooxygenase for enhanced 1-naphthol synthesis and chlorinated ethene degradation. J Bacteriol 184: 344-349.

2. Donegan K, Matyac C, Seidler R, Porteous A (1991) Evaluation of Methods for Sampling, Recovery, and Enumeration of Bacteria Applied to the Phylloplane. Appl Environ Microbiol 57: 51-56.
3. Brown BL, Hadley M, Page R (2008) Heterologous high-level E. coli expression, purification and biophysical characterization of the spine-associated RapGAP (SPAR) PDZ domain. Protein Expr Purif 62: 9-14.
4. Dancheck B, Nairn AC, Peti W (2008) Detailed structural characterization of unbound protein phosphatase 1 inhibitors. Biochemistry 47: 12346-12356.
5. Mustelin T, Tautz L, Page R (2005) Structure of the hematopoietic tyrosine phosphatase (HePTP) catalytic domain: structure of a KIM phosphatase with phosphate bound at the active site. J Mol Biol 354: 150-163.
6. Otwinowski Z, Minor W (1997) Processing of X-ray diffraction data collected in oscillation mode. Macromolecular Crystallography, Pt A 276: 307-326.
7. Pape T, Schneider TR (2004) HKL2MAP: a graphical user interface for macromolecular phasing with SHELX programs. Journal of Applied Crystallography 37: 843-844.
8. Lamzin VS, Wilson KS (1993) Automated Refinement of Protein Models. Acta Crystallographica Section D-Biological Crystallography 49: 129-147.
9. Perrakis A, Sixma TK, Wilson KS, Lamzin VS (1997) wARP: Improvement and extension of crystallographic phases by weighted averaging of multiple-refined dummy atomic models. Acta Crystallographica Section D-Biological Crystallography 53: 448-455.
10. Emsley P, Cowtan K (2004) Coot: model-building tools for molecular graphics. Acta Crystallogr D Biol Crystallogr 60: 2126-2132.
11. Murshudov GN, Vagin AA, Dodson EJ (1997) Refinement of macromolecular structures by the maximum-likelihood method. Acta Crystallogr D Biol Crystallogr 53: 240-255.
12. Painter J, Merritt EA (2006) Optimal description of a protein structure in terms of multiple groups undergoing TLS motion. Acta Crystallogr D Biol Crystallogr 62: 439-450.
13. Lee B, Richards FM (1971) The interpretation of protein structures: estimation of static accessibility. J Mol Biol 55: 379-400.
14. Lawrence MC, Colman PM (1993) Shape complementarity at protein/protein interfaces. J Mol Biol 234: 946-950.
15. Lovell SC, Davis IW, Arendall WB, 3rd, de Bakker PI, Word JM, et al. (2003) Structure validation by Calpha geometry: phi,psi and Cbeta deviation. Proteins 50: 437-450.
16. Vaguine AA, Richelle J, Wodak SJ (1999) SFCHECK: a unified set of procedures for evaluating the quality of macromolecular structure-factor data and their agreement with the atomic model. Acta Crystallogr D Biol Crystallogr 55: 191-205.
17. Terwilliger TC, Berendzen J (1999) Automated MAD and MIR structure solution. Acta Crystallogr D Biol Crystallogr 55: 849-861.
18. Blattner FR, Plunkett G, 3rd, Bloch CA, Perna NT, Burland V, et al. (1997) The complete genome sequence of Escherichia coli K-12. Science 277: 1453-1474.

19. Baba T, Ara T, Hasegawa M, Takai Y, Okumura Y, et al. (2006) Construction of Escherichia coli K-12 in-frame, single-gene knockout mutants: the Keio collection. Mol Syst Biol 2: 2006.0008.
