# Supplementary material for: Three Dimensional Structure of the MqsR:MqsA Complex: A Novel TA Pair Comprised of a Toxin Homologous to RelE and an Antitoxin with Unique Properties
Source: PLoS Pathog. 2009 Dec 24;5(12):e1000706. doi: 10.1371/journal.ppat.1000706 (PMC2791442; doi:10.1371/journal.ppat.1000706)
Supplement: Table S1 — Bacterial strains and plasmids used in this study. (0.05 MB PDF) [file ppat.1000706.s009.pdf]

Table S1. Bacterial strains and plasmids used in this study

Strains and plasmids	Genotype/relevant characteristics	Source
***E. coli* K-12 strains**		
MG1655	F^- λ^- *ilvG rfb*-50 *rph*-1	[18]
BW25113	*lacI*^{q} *rrnB*$_{T14}$ Δ*lacZ*$_{WJ16}$ *hsdR*514 Δ*araBAD*$_{AH33}$ Δ*rhaBAD*$_{LD78}$	[19]
BW25113 *mqsR*	K-12 BW25113 Δ*mqsR* Ω Km^R	[19]
**Plasmids**		
pBS(Kan)	Km^R; cloning vector	[1]
pBS(Kan)-*mqsR*	Km^R; pBS(Kan) P_{lac}::*mqsR*$^+$	this study
pBS(Kan)-*mqsA-F*	Km^R; pBS(Kan) P_{lac}::*mqsA*$^+$	this study
pBS(Kan)-*mqsR-mqsA-F*	Km^R; pBS(Kan) P_{lac}::*mqsR-mqsA*$^+$	this study
pET28a(+)-*mqsR*	Km^R; pET28a(+) P_{T7}::*mqsR*$^+$	this study
pET28a(+)-*mqsA-F*	Km^R; pET28a(+) P_{T7}::*mqsA*$^+$	this study
pET28a(+)-$mqsA_{1\text{-}76}$	Km^R; pET28a(+) P_{T7}::$mqsA_{1\text{-}76}{}^+$	this study
pET28a(+)-$mqsA_{62\text{-}131}$	Km^R; pET28a(+) P_{T7}::$mqsA_{62\text{-}131}{}^+$	this study
pET28a(+)-$mqsA_{62\text{-}131}L81M$	Km^R; pET28a(+) P_{T7}::$mqsA_{62\text{-}131}L81M^+$	this study
pCA21a-*mqsA-F*	Cm^R; pCA21a P_{T7}::*mqsA*$^+$	this study
pCA21a-*mqsA-N*	Cm^R; pCA21a P_{T7}::*mqsA-N*$^+$	this study
pCA21a-*mqsA-C*	Cm^R; pCA21a P_{T7}::*mqsA-C*$^+$	this study

Km^R and Cm^R are kanamycin and chloramphenicol resistance, respectively.
