# Supplementary material for: Three Dimensional Structure of the MqsR:MqsA Complex: A Novel TA Pair Comprised of a Toxin Homologous to RelE and an Antitoxin with Unique Properties
Source: PLoS Pathog. 2009 Dec 24;5(12):e1000706. doi: 10.1371/journal.ppat.1000706 (PMC2791442; doi:10.1371/journal.ppat.1000706)
Supplement: Table S2 — Oligonucleotides used for this study. (0.02 MB PDF) [file ppat.1000706.s010.pdf]

Table S2. Oligonucleotides used for this study. f indicates forward primer and r indicates reverse primer.

Name	Sequence
**Construction of plasmids**	
pBS(Kan)-*mqsR*	f: 5’-GCGCGCGATCGGATCCACTAAAGTAACAGGGAGGCGGGGGTTATG-3’<br>r: 5’-GCGCGCCTAGTCTAGATGGCAAACCGGACATTTCATATTACTTC-3’
pBS(Kan)-*mqsA-F*	f: 5’-GCGCGCGATCGGATCCAGGCCAGGTTTATCTTAA AATTACGGTA-3’<br>r: 5’-GCGCGCCTAGTCTAGACCCGCTTTTCCATTAATTAACGGATTTC-3’
pBS(Kan)-*mqsR-mqsA-F*	f: 5’-GCGCGCGATCGGATCCACTAAAGTAACAGGGAGGCGGGGGTTATG-3’<br>r: 5’-GCGCGCCTAGTCTAGACCCGCTTTTCCATTAATTAACGGATTTC-3’
**EMSA**	
P*mqsR*	f: 5’-GTGATGCCTGACTCCAGCTT-3’<br>r: 5’-CGTGTATGTGGTGTGCGTTT-3’
P*mcbR*	f: 5’-GCAAAGTGGTGATCCGCG-3’<br>r: 5’-CCTGCAGAGTCAAACTGA-3’
P*spy*	f: 5’-CAGTCATCCGGTATAGTT-3’<br>r: 5’-GGCAACAAACAGTGCAGT-3’
P*tomB*	f: 5’-CGGGTTAGTGCTAGTATGAAAAAGT-3’<br>r: 5’-ACTTAAGCTGTGCGATATCATGTCT-3’
