# Supplementary material for: Three Dimensional Structure of the MqsR:MqsA Complex: A Novel TA Pair Comprised of a Toxin Homologous to RelE and an Antitoxin with Unique Properties
Source: PLoS Pathog. 2009 Dec 24;5(12):e1000706. doi: 10.1371/journal.ppat.1000706 (PMC2791442; doi:10.1371/journal.ppat.1000706)
Supplement: Table S3 — Data collection and refinement statistics for MqsA-N. (0.05 MB PDF) [file ppat.1000706.s011.pdf]

Table S3. Data Collection and Refinement Statistics for MqsA-N

	MqsA-N (peak)	MqsA-N (remote)
**Data Collection[1]**		
Space Group	P 2_1 2_1 2_1	
Unit cell (Å)	30.8, 52.1, 53.8	
Wavelength (Å)	0.9787	0.9321
Resolution (Å)	50.0-1.70 (1.73-1.70)	50.0-1.70 (1.73-1.70)
R_{sym} (%)	6.4 (31.4)	5.7 (26.2)
<I/σI>	27.25 (7.98)	21.10 (6.62)
Completeness (%)	97.5 (97.0)	97.4 (96.7)
Redundancy	8.8 (9.1)	5.0 (5.2)
**Refinement Statistics**		
Resolution (Å)		37.42-1.70
R_{cryst} (%)		16.5
R_{free} (%)		18.6
Protein atoms		534
Waters		97
Ligand/Ion atoms		11
r.m.s.d bond length (Å)		0.012
r.m.s.d bond angle (°)		1.283
**Average B factors ($Å^2$)**		
Protein		15.80
Water		34.73
Ligand/ions		32.44
**Ramachandran Plot**		
Favored (%)		98.6
Allowed (%)		1.4
Disallowed (%)		0.0
**PDB Code**		3GA8

[1] Highest-resolution shell data are shown in parentheses
