# Supplementary material for: Three Dimensional Structure of the MqsR:MqsA Complex: A Novel TA Pair Comprised of a Toxin Homologous to RelE and an Antitoxin with Unique Properties
Source: PLoS Pathog. 2009 Dec 24;5(12):e1000706. doi: 10.1371/journal.ppat.1000706 (PMC2791442; doi:10.1371/journal.ppat.1000706)
Supplement: Table S4 — Data collection and refinement statistics for MqsA-C. (0.05 MB PDF) [file ppat.1000706.s012.pdf]

Table S4. Data Collection and Refinement Statistics for MqsA-C

	MqsA-C High Resolution	MqsA-C (peak)	MqsA-C (inflection)	MqsA-C (remote)
**Data Collection**[1]				
Space Group	P 3_1 2 1		P 3_1 2 1	
Unit Cell (Å)	39.6, 39.6, 78.3		39.35 39.35 78.3	
Wavelength (Å)	1.0	0.9788	0.9794	0.9322
Resolution (Å)	50.0-1.40 (1.42-1.40)	50.0-2.0 (2.07-2.0)	50.0-2.0 (2.07-2.0)	50.0-2.0 (2.07-2.0)
R_{sym} (%)	3.2 (23.2)	4.3 (6.8)	4.0 (6.5)	4.1 (7.5)
**<I/σI>**	31.5 (6.19)	77.1 (50.6)	53.8 (34.7)	28.8 (18.5)
Completeness (%)	99.6 (99.2)	99.7 (97.0)	99.6 (96.4)	99.9 (99.6)
Redundancy	5.7 (4.5)	8.5 (7.3)	4.2 (3.7)	4.3 (4.3)
**Refinement Statistics**				
Resolution (Å)	20.77-1.40			
R_{cryst} (%)	15.5			
R_{free} (%)	18.2			
Protein atoms	571			
Waters	80			
r.m.s.d bond length (Å)	0.011			
r.m.s.d bond angle (°)	1.413			
**Average B factors ($Å^2$)**				
Protein	15.01			
Water	29.33			
Ligand/ions	N/A			
**Ramachandran Plot**				
Favored (%)	100.0			
**PDB Code**	3FMY			

[1] Highest-resolution shell data are shown in parentheses
